# Efficacy and Acceptability of Glycemic Control of Glucagon-Like Peptide-1 Receptor Agonists among Type 2 Diabetes: A Systematic Review and Network Meta-Analysis

**DOI:** 10.1371/journal.pone.0154206

**Published:** 2016-05-09

**Authors:** Zhixia Li, Yuan Zhang, Xiaochi Quan, Zhirong Yang, Xiantao Zeng, Linong Ji, Feng Sun, Siyan Zhan

**Affiliations:** 1 Department of Epidemiology and Biostatistics, School of Public Health, Peking University Health Science Center, Beijing, China; 2 Department of Clinical Epidemiology and Biostatistics, McMaster University, 1280 Main Street West, Hamilton, ON, Canada; 3 Center for Evidence-based and Translational Medicine, Zhongnan Hospital, Wuhan University, Wuhan, China; 4 Department of Endocrinology and Metabolism, People’s Hospital, Peking University, Beijing, China; Baylor College of Medicine, UNITED STATES

## Abstract

**Objective:**

To synthesize current evidence of the impact of Glucagon-like peptide-1 receptor agonists (GLP-1 RAs) on hypoglycemia, treatment discontinuation and glycemic level in patients with type 2 diabetes.

**Design:**

Systematic review and network meta-analysis.

**Data Sources:**

Literature search (Medline, Embase, the Cochrane library), website of clinical trial, bibliographies of published systematic reviews.

**Eligibility Criteria:**

Randomized controlled trials with available data comparing GLP-1 RAs with placebo or traditional anti-diabetic drugs in patients with type 2 diabetes.

**Data Synthesis:**

Traditional pairwise meta-analyses within DerSimonian-Laird random effects model and network meta-analysis within a Bayesian framework were performed to calculate odds ratios for the incidence of hypoglycemia, treatment discontinuation, HbA1c<7.0% and HbA1c<6.5%. Ranking probabilities for all treatments were estimated to obtain a treatment hierarchy using the surface under the cumulative ranking curve (SUCRA) and mean ranks.

**Results:**

78 trials with 13 treatments were included. Overall, all GLP-1 RAs except for albiglutide increased the risk of hypoglycemia when compared to placebo. Reduction in the incidence of hypoglycemia was found for all GLP-1 RAs versus insulin (except for dulaglutide) and sulphonylureas. For the incidence of treatment discontinuation, increase was found for exenatide, liraglutide, lixisenatide and taspoglutide versus placebo, insulin and sitagliptin. For glycemic level, decrease was found for all GLP-1 RAs versus placebo. Dulaglutide, exenatide long-acting release (exe_lar), liraglutide and taspoglutide had significant lowering effect when compared with sitagliptin (HbA1c<7.0%) and insulin (HbA1c<6.5%). Finally, according to SUCRAs, placebo, thiazolidinediones and albiglutide had the best decrease effect on hypoglycemia; sulphanylureas, sitagliptin and insulin decrease the incidence of treatment discontinuation most; exe_lar and dulaglutide had the highest impact on glycemic level among 13 treatments.

**Conclusions:**

Among 13 treatments, GLP-1 RAs had a significant reduction with glycemic level but a slight increase effect on hypoglycemia and treatment discontinuation. While albiglutide had the best decrease effect on hypoglycemia and treatment discontinuation among all GLP-1 RAs. However, further evidence is necessary for more conclusive inferences on mechanisms underlying the rise in hypoglycemia.

## Introduction

An increasing number of patients with type 2 diabetes mellitus (T2DM) are being treated with glucagon-like peptide-1 receptor agonists (GLP-1 RAs), a new class of anti-diabetic agents based on incretin therapy[1, 2]. GLP-1 RAs are analogues of GLP-1, which could stimulate insulin secretion, improve insulin resistance and slow down gastrointestinal motility [3–5]. Exenatide (Byetta; Eli Lilly & Co.), liraglutide (Victoz; Novo Nordisk), the two earliest GLP-1 RAs, were approved by the United States Food and Drug Administration (FDA) in 2005 and 2010, respectively [6, 7]. Albiglutide (Tanzeum/Eperzan, GSK) and lixisenatide (Lyxumia, Sanofi) were approved by European Medical Agency (EMA) in 2013. Recently, Dulaglutide (Trulicity; Eli Lilly & Co.) was approved by FDA in 2014. Taspoglutide is currently in phase III clinical trials.

According to the International Diabetes Federation (IDF) in 2013, 387 million people are currently diagnosed with diabetes and there is a projected rise to 592 million people in the world living with diabetes by the year 2035[8]. It means that more and more people will need to be prescribed anti-diabetes medication to help achieve the recommended HbA1c target of <6.5% (National Institute for Health and Clinical Excellence (NICE), 2008) or HbA1c target of <7.0% (American Diabetes Association, (ADA))[9] to avoid the devastating complications of poor diabetes control. Patients with poorly controlled glycemic level would greatly increase the risk of hypoglycemia [10–12] and treatment discontinuation [13–15]. Therefore, an ideal anti-diabetic treatment would be one that can couple the achievement of glycemic control with a low propensity for causing hypoglycemia and treatment discontinuation. Indeed, several clinical trials and meta-analyses[16–21] for GLP-1 RAs have demonstrated the lowering effect of glycemic levels as well as raised hypoglycemia and treatment discontinuation, although the mechanisms are not very clearly understood. However, since there are so much medicines to choose, which is better for clinical decision is still unknown. So there is a need to include all kinds of GLP-1 RAs simultaneously to assess the impact on hypoglycemia and treatment discontinuation between any two of them.

Therefore, we collected all randomized controlled trials (RCTs) of comparing GLP-1 RAs with placebo or traditional anti-diabetic drugs. A conventional pairwise meta-analysis was performed to summarize current evidence for the effect of GLP-1 RAs on hypoglycemia, treatment discontinuation and glycemic level in patients with T2DM. Additional network meta-analysis was conducted to assess the robustness of the pairwise meta-analysis, supplement missing evidence of head-to-head comparisons by combining both direct and indirect evidence and rank treatments in the evidence network.

## Method

### Systematic review registration

PROSPERO register, CRD42014015328

### Search strategy

In consultation with a medical librarian, a search strategy for MEDLINE, EMBASE and the Cochrane library (from inception to June 1st, 2014) was established. The following search strategy for Ovid-MEDLINE was adapted for other databases:

1. exp glucagon-like peptide-1 agonists/
2. (glucagon like peptide* or GLP-1).tw.
3. (exenatide or liraglutide or albiglutide or taspoglutide or lixisenatide or LY2189265).tw.
4. randomized controlled trial.pt.
5. (randomized or randomised).tw.
6. (1 or 2 or 3) and (4 or 5)

In addition, completed but unpublished trials were identified from www.clinicaltrials.gov website using the similar search strategy. The bibliographies of published systematic reviews were also searched. All relevant authors and principal manufacturers were contacted to supplement incomplete reports of the original papers or to provide new data for unpublished studies.

### Study selection

All the studies included are in English and they are eligible for inclusion only if they were RCTs involving GLP-1 RAs, active anti-diabetic drugs or placebo with complete data on hypoglycemia, treatment discontinuation or glycemic level. Trials are excluded if only they meet one of the following: (1) trials are not RCT (e.g., review, expert comment, editor opinion, new agent introduction, single case report, or case series); (2) if several studies included the same clinical trial, we only include the one which had the longest follow-up time and excluded the other early studies; (3) experimentation on animals or in vitro; (4) not conducted in T2DM; (5) pharmacokinetics research; (6) trials underway, unfinished, or suspended; (7) economical evaluation research; (8) other unrelated researches. These studies were approved by the local ethics committees and written informed consent was obtained from all the patients. The eligibility of studies for inclusion criteria was assessed independently by four reviewers (ZXL, YZ, XCQ and ZRY) in duplicate.

### Data extraction and quality evaluation

Data were extracted using ADDIS software[22] with respect to trial information (author, publication year, sample size, trial duration, types of intervention and control), population characteristics (background therapy, diabetes duration, age, baseline level of HbA1c), reported outcomes (Number of hypoglycemia, treatment discontinuation, HbA1c<7.0% and HbA1c<6.5% events in each group) and information on methodology. Four investigators (ZXL, ZRY, XCQ and XTZ) extracted data independently, in duplicate. Any discrepancies were resolved by consensus between the two independent reviewers or by a senior investigator (FS).

Quality of studies was assessed according to JADAD scale[23], including adequate method for randomization, appropriate blinding procedures, and detailed report of withdrawals. The JADAD score was not used as a selection criterion, but only for descriptive purpose.

### Data analysis

#### Methods for direct treatment comparisons

Traditional pairwise meta-analyses was performed using DerSimonian-Laird random effects model[24]. Odds ratio (OR) for hypoglycemia, treatment discontinuation, HbA1c<7.0% and HbA1c<6.5% with 95% confidence interval (CI) were calculated as effect measures. For studies that did not report intention-to-treat, we analyzed outcomes as all-patients randomized. The *I*^*2*^-statistic was calculated as a measure of the proportion of the overall variation that is attributable to between-study heterogeneity[25].

#### Methods for indirect and mixed comparisons

A random-effects network meta-analysis within a Bayesian framework[26] was performed to evaluate the relative effectiveness of each kind of GLP-1 RAs on hypoglycemia, HbA1c<7.0%, HbA1c<6.5% and the relative acceptability on treatment discontinuation. Bayesian network meta-analysis is a generalization of traditional meta-analysis that allows all evidence to be taken into account simultaneously (both direct and indirect). It can be applied whenever a connected network of evidence is available[26]. ORs for hypoglycemia, treatment discontinuation, HbA1c<7.0% and HbA1c<6.5% with 95% credible interval (CrI) were summarized. The posterior densities for all unknown parameters were estimated using MCMC (Markov chain Monte Carlo) for each model. Each chain used 40 000 iterations with a burn-in of 20 000.

Network meta-analyses enable estimation of the probability that each intervention is the best for each outcome. Probabilities for each treatment taking each possible rank were plotted in absolute rankograms or cumulative rankograms. Besides, the surface under the cumulative ranking curve (SUCRA)[27] were used to estimate the ranking probabilities for all treatments in order to obtain a treatment hierarchy. SUCRA is a percentage interpreted as the percentage of efficacy of a treatment on the outcome that would be ranked first without uncertainty, which is equal to 1 when the treatment is certain to be the best and 0 when it is certain to be the worst[27].

An absolute measure of fit ${\bar{\text{D}}}_{\text{res}}$, was considered to formally check the model’s overall fit. ${\bar{\text{D}}}_{\text{res}}$ is the posterior mean of the residual deviance (the deviance for the fitted model minus the deviance for the saturated model). Ideally, each data point should contribute about one to the posterior mean deviance so that it can be compared to the number of data points for the purpose of checking model fit[28].

Loop-specific approach was used to evaluate the presence of inconsistency locally in network meta-analysis models, that is, if the information of both sources of evidence is similar enough to be combined[29]. This method evaluates the consistency assumption in each closed loop of the network separately. Difference (inconsistency factor) with 95% CIs between direct and indirect estimations for a specific comparison was calculated to assess the presence of inconsistency in each loop. Inconsistency was defined as disagreement between direct and indirect evidence with a 95% CI excluding 0.

Analyses were conducted using STATA 11.0 (pairwise meta-analysis, *I*^*2*^ calculations and estimation of inconsistency), R 3.0.2 (SUCRA graphs) and WinBUGS 1.4.3 (network Meta-analysis and model fit).

## Results

### Study characteristics and evidence network

**Fig 1:**
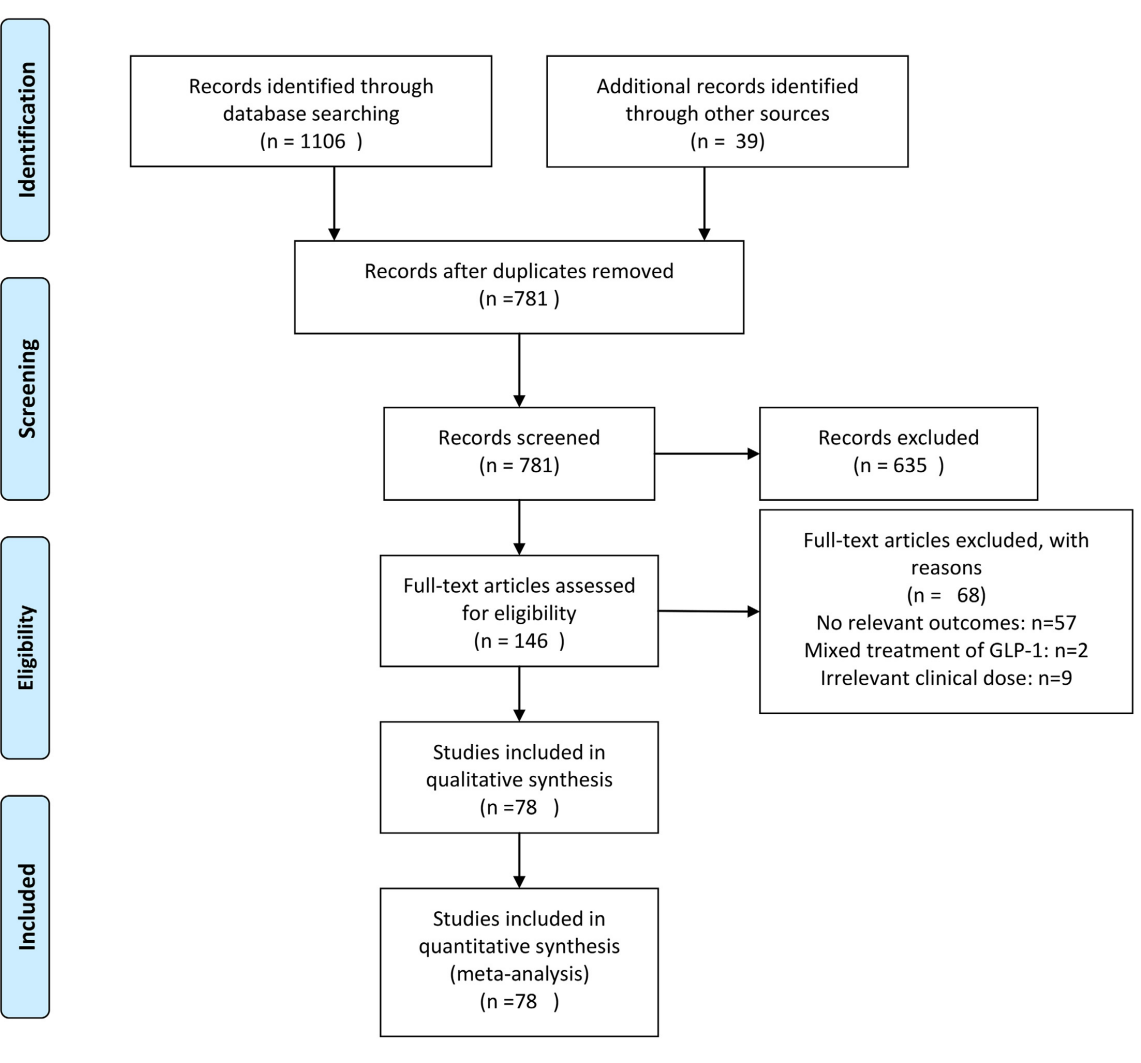
Flow chart of studies considered for inclusion, RCT = randomized controlled trial. This Flow chart is based on PRISMA 2009 Flow Diagram[30].

78 trials involving 13 treatments met the selection criteria. A total of 34685 patients contributed to the analysis. Flow chart of trials selection was shown in Fig 1.

#### Study characteristics

Table 1 summarized the characteristics of included 78 trials. The range of publication year was 2004–2014. Trial duration ranged from 26 to 234 weeks. The average age of included patients was 55.89 years [standard deviation (SD): 1.71], varied from 50.5 to 61.0 years. The median diabetes duration at baseline was 7.5 [interquartile range (IQR): 6.0–8.9] years. And the mean baseline glycemic level HbA1c was 8.2% (SD: 0.4%). Of the 78 trials included, albiglutide, dulaglutide, exenatide long-acting release (exe_lar), exenatide, liraglutide, lixisenatide and taspoglutide were studied in 3, 3, 7, 25, 13, 9 and 8 trials, respectively. And 3 trials involved both exenatide and exe_lar simultaneously. Besides, albiglutide and exenatide, albiglutide and liraglutide, dulaglutide and exenatide, exenatide and taspoglutide, exenatide and lixisenatide, liraglutide and exenatide, liraglutide and exe_lar were both involved simultaneously in 1 trial, respectively.

**Table 1:** Characteristics of the 78 studies with 34685 patients included in the network Meta-analysis Duration > = 8w.

NO	Study ID	Investigational treatments	Outcome reported	Size (pts)	Background therapy	Duration of trial(w)	Baseline information		
							Age (yrs)	Years of T2DM	HbA1c(%)
1	Ahren B, 2013[31]	Placebo,Lixisenatide	1, 2, 3, 4	680	Met	24	54.8	3.6	8.1
2	Ahren B, 2014[32]	Albiglutide,SU, placebo, Sitagliptin	1, 2, 3, 4	1012	Met	104	54.5	6	8.11
3	Apovian CM, 2010[33]	Exenatide,Placebo	1, 2	194	Met/SU/SU+Met	24	54.8	5.5	7.6
4	Barnett AH, 2007[34]	Exenatide,Insulin	1, 3, 4	276	Met/SU	16	54.9	7.4	9
5	Bergenstal R, 2009[35]	Exenatide,Insulin	1, 3, 4	372	Met+SU	24	52.2	8.5	10.2
6	Bergenstal RM, 2010 [36]	Sitagliptin,TZD,Exe_LAR	1, 2, 3, 4	491	Met	26	52.5	6	8.6
7	Bergenstal RM, 2012[37]	Placebo,Sitagliptin,Taspoglutide	1, 2, 3	546	Met	24	56	5.9	8
8	Blevins T, 2011[38]	Exenatide,Exe_LAR	1, 2, 3, 4	252	Met+/-SU+/-TZD	24	55.5	7	8.45
9	Bolli G, 2013[39]	Placebo,Lixisenatide	1, 2, 3, 4	479	Met	24	56.1	6	8
10	Bunck MC, 2009[40]	Exenatide,Insulin	1, 3	69	Met	52	58.3	4.9	7.5
11	Buse JB, 2004[41]	Exenatide,Placebo	1, 3	377	SU	30	55	6.3	8.6
12	Buse JB, 2009[42]	Liraglutide,Exenatide	1, 2, 3, 4	464	Met+/-SU	26	56.7	8.2	8.2
13	Buse JB, 2011[43]	Exenatide,Placebo	1, 2, 3, 4	259	Glar+/-Met/TZD	30	59	12	8.4
14	Buse JB, 2013[44]	Liraglutide,Exe_LAR	1, 2, 3	911	Met+/-SU+/-Piog	26	59	12	8.4
15	Charbonnel B, 2013[45]	Liraglutide,Sitagliptin	1, 2, 3, 4	650	Met	26	57.3	6	8.2
16	Davies M, 2013[46]	Exe_LAR,Insulin	1, 2, 3, 4	216	Met/Met+SU	26	58.5		8.4
17	Davies MJ, 2009[47]	Exenatide,Insulin	1, 3, 4	234	Met+/- SU/TZD	26	56.5	8.7	8.6
18	Davis SN, 2007[48]	Exenatide,Insulin	1, 3	49	SU/Met	16	53	11	8.1
19	DeFronzo RA, 2005[49]	Exenatide,Placebo	1, 3	336	Met	30	53	5.8	8.2
20	DeFronzo RA, 2010[50]	Exenatide,TZD	1	90	Met	20	56	4.7	7.8
21	Derosa G, 2012[51]	Exenatide,Placebo	1, 2	171	Met	48	57	7.7	8
22	Diamant M, 2012[52]	Exe_LAR,Insulin	1, 2, 3, 4	466	Met/Met+SU	26	58	7.9	8.3
23	Drucker DJ, 2008[53]	Exenatide,Exe_LAR	1, 2, 3	295	Met+/-SU+/-TZD	30	55	6.5	8.3
24	Fonseca VA, 2012[54]	Placebo,Lixisenatide	1, 2, 3, 4	361	no	12	53.6	1.3	8
25	Gallwitz B, 2011[55]	Exenatide,Insulin	1, 3, 4	354	Met/SU	26	57	5	7.9
26	Gallwitz B, 2012[56]	SU,Exenatide	1, 2	1019	Met	234	56	5.6	7.45
27	Gao Y, 2009[57]	Exenatide,Placebo	1	466	Met/Met+SU	16	54.5	8	8.3
28	Garder A, 2011[58]	SU,Liraglutide	1, 3, 4	745	Met/SU/BG/Met+TZD	104	53	5.4	8.3
29	Heine RJ, 2005[59]	Exenatide,Insulin	1	549	Met+SU	26	58.9	9.6	8.2
30	Henry RR, 2013[60]	Placebo,Taspoglutide	1, 2, 3, 4	320	Met+Piog	24	54.1	7.7	8.1
31	Hollander P, 2013[61]	Placebo,Taspoglutide	1, 2, 3, 4	292	Met	24	53.5	5.08	7.55
32	Inagaki N, 2012[62]	Exe_LAR,Insulin	1, 3, 4	427	BG or BG + TZD	26	56.76	9.03	8.5
33	Iwamoto K, 2009[63]	Placebo,Exe_LAR	3, 4	19	SU/BG/SU+BG/TZD	10	58	6	7.4
34	Ji LN, 2013[64]	Exenatide,Exe_LAR	2, 3, 4	678	Met, SU, TZD	26	55	8.2	8.7
35	Kadowaki T, 2009[65]	Exenatide,Placebo	1, 3	114	SU/BG/SU+TZD/BG	12	60.3	11.8	8
36	Kadowaki T, 2011[66]	Placebo,Exenatide	1, 3, 4	179	SU+/BG+/TZD	24	58.4	12	8.2
37	Kendall DM, 2005[67]	Exenatide,Placebo	1, 3	743	Met/Met+SU	30	55.3	8.9	8.5
38	Kim D, 2007[68]	Placebo,Exe_LAR	1, 2, 3	29	Met	15	54	5	8.5
39	Li CJ, 2012[69]	Liraglutide,Insulin	1	84	Insulin	12	52	9	8.75
40	Liutkus J, 2010[70]	Exenatide,Placebo	1, 3, 4	165	TZD/TZD+Met	26	54.7	6.4	8.2
41	Marre M, 2009[71]	Liraglutide,Placebo,TZD	1, 2, 3, 4	808	Glimepiride	26	56.1	6.5	8.4
42	Mathieu C, 2014[72]	Liraglutide,Insulin	2, 3	177	Insulin degludec (IDeg) OD + Met	52	61	12, 4	7.7
43	Moretto TJ, 2008[73]	Placebo,Exenatide	1, 2, 3, 4	232	diet+exercise	24	54	1.7	7.8
44	Nauck M, 2014[74]	Dulaglutide, placebo, Sitagliptin	1, 2, 3, 4	921	diet+exercise/monotherapy /Met+monotherapy	26/52	54	7	8.1
45	Nauck MA, 2007[75]	Exenatide,Insulin	1, 2, 3	501	Met/SU	52	58.5	9.9	8.6
46	Nauck MA, 2009[76]	Placebo,Taspoglutide	2, 3	197	Met	8	54	5.5	7.9
47	Nauck M, 2009[77]	SU,Liraglutide,Placebo	1, 2, 3, 4	845	Met	104	57	7.9	8.4
48	Nauk M, 2013[78]	Insulin,Taspoglutide	1, 2, 3, 4	1028	Met/SU	24	58	9.1	8.3
49	NCT00620282, 2011[79]	SU,Placebo,Liraglutide	1	49	Met	12	58.5	6.8	7.2
50	NCT00667732, 2013[80]	Exenatide,Placebo	1, 4	34	Met+/-Lantus Insulin				
51	NCT00701935, 2013[81]	Exenatide,Placebo	3	71		24	58		
52	Pinget M, 2013[82]	Placebo,Lixisenatide	1, 3, 4	484	Piog+/-Met	24	55.5	1.75	8.1
53	Pratley R, 2011[83]	Liraglutide,Sitagliptin	1, 3, 4	658	Met	52	55.3	6.2	8.4
54	Pratley RE, 2013[84]	Taspoglutide,TZD	1, 3, 4	740	Met/SU/Met+SU	24	56.4	8.8	8.3
55	Pratley RE, 2014[85]	Albiglutide,Liraglutide	1, 2, 3, 4	805		32	55.6	8.35	8.17
56	Ratner R, 2010[86]	Placebo, Taspoglutide	1, 2, 3, 4	129	Met	8	56.5	6.5	7.9
57	Ratner RE, 2010[87]	Placebo,Lixisenatide	1, 3, 4	529	Met	13	56.5	7.1	7.5
58	Raz I, 2012[88]	Placebo,Taspoglutide	1, 2, 3, 4	354	no	24	54.8	2.4	7.6
59	Riddle MC,2013[89]	Placebo,Lixisenatide	1, 2, 3, 4	446	Insulin Glar + Met	24	56	9.2	7.6
60	Riddle MC, 2013[90]	Placebo,Lixisenatide	1, 2, 3, 4	495	Met+/-SU+/-TZD	24	57	12.5	8.4
61	Rosenstock J, 2013[91]	Exenatide,Taspoglutide	1, 2, 3	1149	TZD+/-Met	24	56	6.6	8.1
62	Rosenstock J, 2014[92]	Placebo,Lixisenatide	1, 2, 3	859	Met+/-SU	24	57.2	9.4	8.3
63	Rosenstock J, 2014[93]	Albiglutide, Insulin	1, 2, 3, 4	566	Insulin Glar	26	55.45	11	8.45
64	Rosenstock J, 2009[94]	Albiglutide,Exenatide,Placebo	1, 3	214	Met	16	54	4.9	8
65	Rosenstock J, 2013[95]	Exenatide,Lixisenatide	1, 2, 3, 4	634	Met	24	57.4	6.8	8
66	Russell-Jones D, 2009[96]	Placebo,Liraglutide,Insulin	1, 2, 3	576	Met+Glimepiride	26	57.6	9.5	8.3
67	Russell-Jones D, 2012[97]	Sitagliptin,Metformin,TZD,Exe_LAR	1, 3, 4	820	diet + exercise	26	53.8	2.7	8.5
68	Seino Y, 2008[98]	Liraglutide,Placebo	1, 2, 3, 4	211	oad	14	57	8	8.3
69	Seino Y, 2010[99]	SU,Liraglutide	3, 4	400	diet+exercise/monotherapy	52	58.3	8.3	8.9
70	Seino Y, 2012[100]	Placebo,Lixisenatide	1, 2, 3, 4	311	Insulin/SU	24	58.4	13.9	8.53
71	Seino Y, 2014[101]	Albiglutide, placebo	2	211	diet+exercise/monotherapy	16	57	7	8.55
72	Umpierrez G, 2014[102]	Dulaglutide, Met	1, 2, 3, 4	807	diet + exercise	26/52	56	3	7.6
73	Umpierrez GE, 2011[103]	Placebo,LY	1, 2	262	oad	16	56.5	8.3	8.3
74	Wysham C, 2014[104]	Dulaglutide, placebo, Exenatide	1, 2, 3, 4	976	Met+Piog	26	55.57	9	8.1
75	Yang W, 2011[105]	SU,Liraglutide	1, 2, 3	928	Met	16	53.3	7.5	8.6
76	Yuan GH, 2012[106]	Exenatide,Metformin	1, 3, 4	59	no	26	50.5	<1 month	8.2
77	Zinman B, 2007[107]	Exenatide,Placebo	1, 3	233	TZD+/-Met	16	56	8	7.9
78	Zinman B, 2009[108]	Liraglutide,Placebo	1, 2, 3, 4	533	Met+Rosig	26	55	8.9	8.5

Note: LY: LY2189265, Dulaglutide. SU: sulphanylureas; TZD: thiazolidinedione; MET: metformin; Glar: glargine; Piog: pioglitazone; BG: biguanide; Rosig: rosiglitazone; oad: oral antidiabetic drug;—: unavailable information.

1: Hypoglycemia, 2: treatment discontinuation, 3:HbA1c<7.0%, 4: HbA1c<6.5%

Reporting quality of included studies varied. According to JADAD scale, the number of dropout and the methods used for randomization, allocation concealment and blinding were appropriately described in most cases (89.7%, 85.9%, 60.3% and93.6%, respectively), although 34.6% (27/78) of trials were open label. Additionally, 88.5% (69/78) of trials used intention-to-treat analysis. (S1 Table). Overall, risk of bias is respectively low.

#### Evidence network

**Fig 2:**
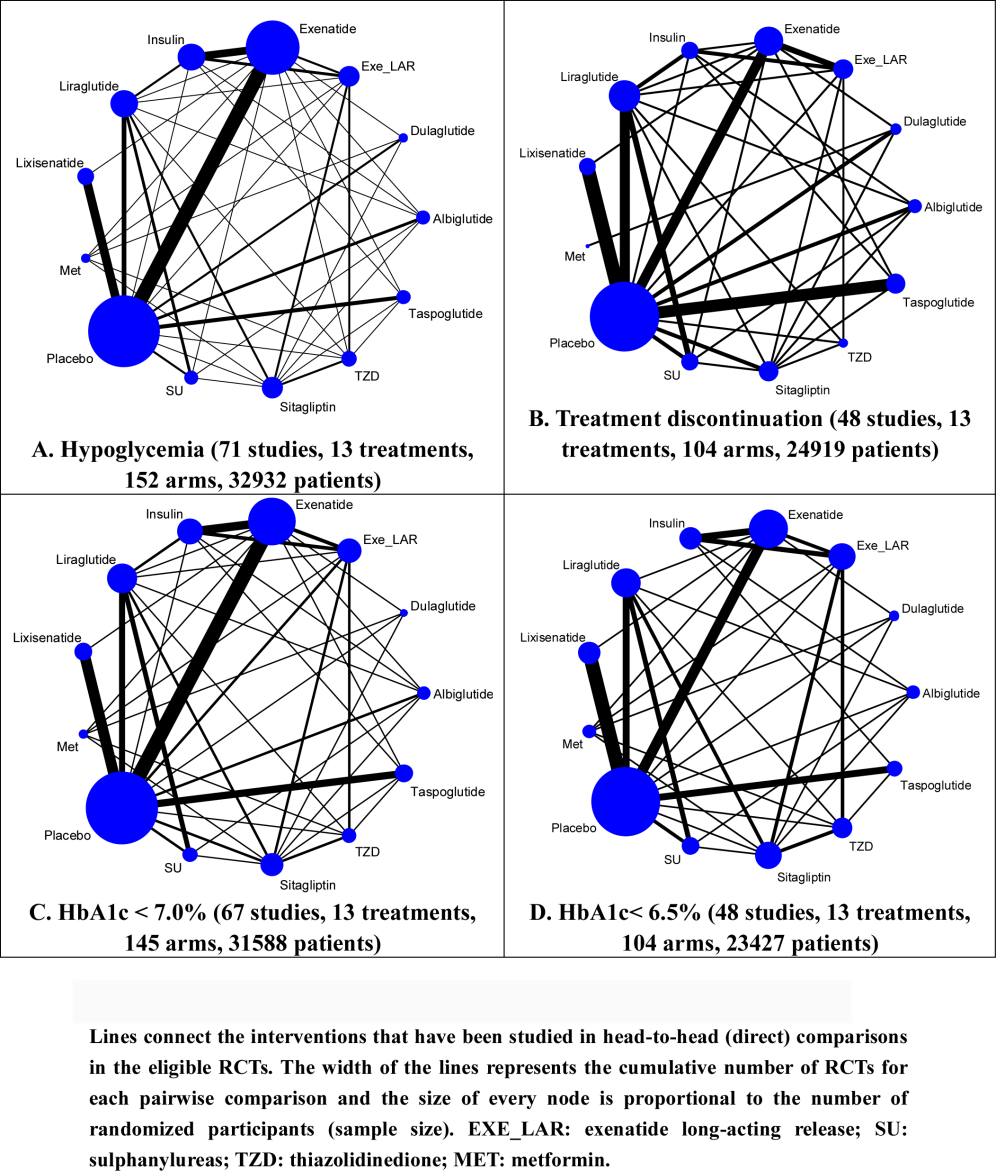
Evidence structure of eligible comparisons for network meta-analysis.

13 treatments were analyzed, including 7 GLP-1 RAs (Albiglutide, Dulaglutide, Exe_LAR, Exenatide, Liraglutide, Lixisenatide and Taspoglutide), 5 kinds of active anti-diabetic drugs (insulin, metformin (Met), sulphonylureas (SU), sitagliptin and thiazolidinediones (TZD)), and placebo. 85.90% (67/78) of trials were two-arm studies and the rest 14.10% (11/78) were multiple-arm studies (see Table 1 and Fig 2). Overall, 32932, 24919, 31588 and 23427 patients contributed to the analysis of hypoglycemia (Fig 2A, including 71 studies and 13 treatments), treatment discontinuation (Fig 2B, including 48 studies and 13 treatments), HbA1c < 7.0% (Fig 2C, including 67 studies and 13 treatments) and HbA1c < 6.5% (Fig 2D, including 48 studies and 13 treatments), respectively. Every group of GLP-1 RAs existed head-to-head (direct) comparison with placebo.

#### Conventional meta-analysis of individual GLP-1 RAs

**Fig 3:**
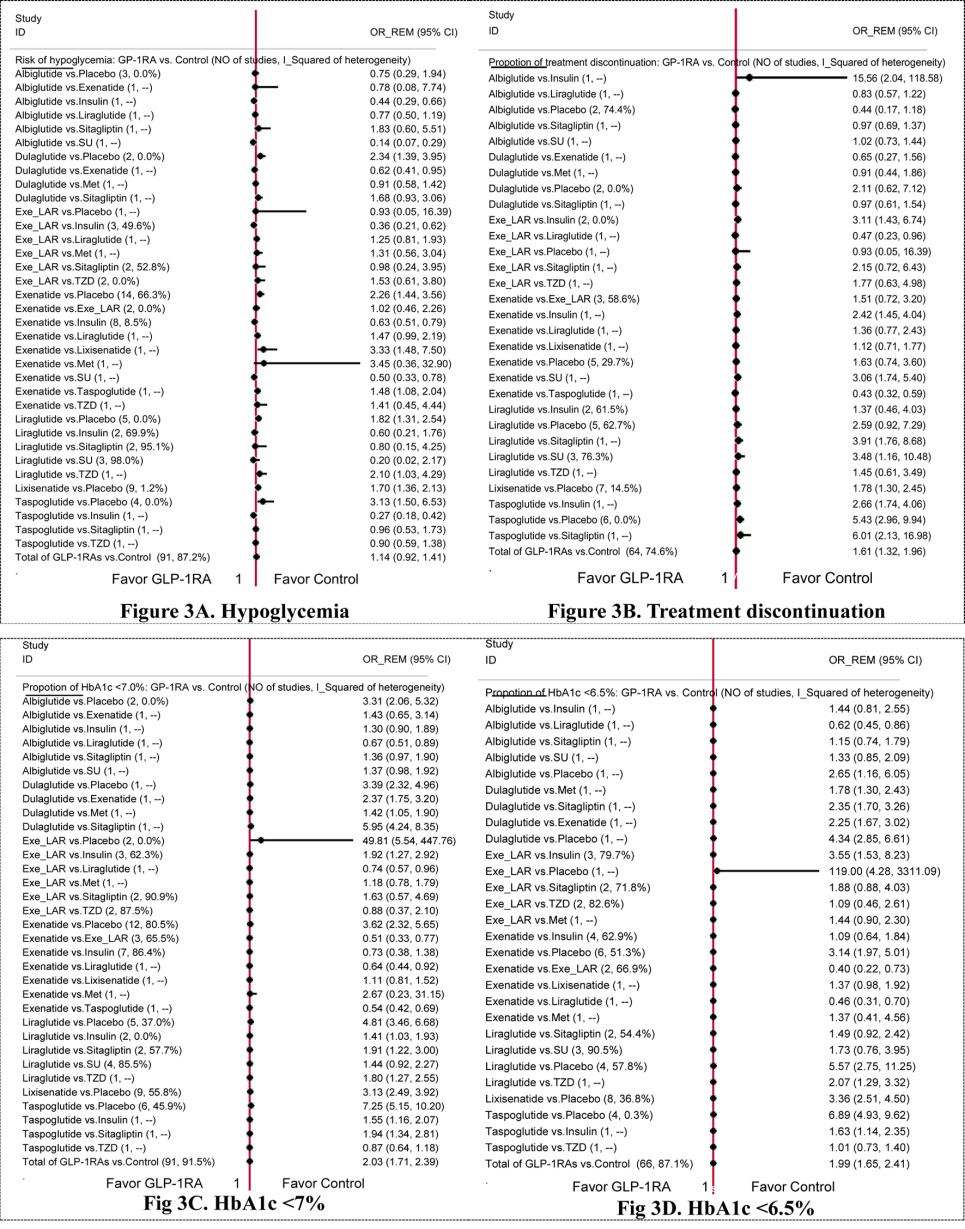
Impact of individual GLP-1 receptor agonists on hypoglycemia, treatment discontinuation, HbA1c <7.0%, HbA1c <6.5% of direct pairwise meta-analysis.

Fig 3A–3D showed the effect of individual GLP-1 RAs on hypoglycemia, treatment discontinuation, HbA1c<7.0% and HbA1c<6.5% from direct pairwise meta-analysis.

Fig 3A displayed the effect on hypoglycemia. In comparison to placebo, dulaglutide, exenatide, liraglutide, lixisenatide and taspoglutide significantly increased the risk of hypoglycemia by 2.34 (95% CI: 1.39, 3.95), 2.26 (95%CI: 1.44, 3.56), 1.82 (95%CI: 1.31, 2.54), 1.70 (95%CI: 1.36, 2.13) and 3.13 (95%CI: 1.50, 6.53), respectively. No significant difference was found between albiglutide or exe_lar versus placebo. Compared with insulin, exe_lar (OR 0.36, 95%CI: 0.21, 0.62) and exenatide (OR 0.63, 95%CI: 0.51, 0.79) were associated with less hypoglycemia. No statistically significant difference was found between GLP-1 RAs and other active comparators in their effects on hypoglycemia.

Regarding treatment discontinuation, lixisenatide and taspoglutide significantly increased the incidence in comparison to placebo by 1.78 (95%CI: 1.30, 2.45) and 5.43 (95%CI: 2.96, 9.94), respectively. Significant increase was also found when exe_lar versus insulin (OR 3.11 (95%CI: 1.43, 6.74)) and liraglutide versus SU (OR 3.48 (95%CI: 1.16, 10.48)). No statistically significant difference was found between other GLP-1 RAs versus placebo or active comparators in their effects on treatment discontinuation (Fig 3B).

Fig 3C displayed the effect on HbA1c <7%. In comparison to placebo, albiglutide, exe_lar, exenatide, liraglutide, lixisenatide and taspoglutide significantly increased the incidence of HbA1c <7% by 3.31 (95% CI: 2.06, 5.32), 49.81 (95%CI: 5.54, 447.76), 3.62 (95%CI: 2.32, 5.65), 4.81 (95%CI: 3.46, 6.68), 3.13 (95%CI: 2.49, 3.92) and 7.25 (95%CI: 5.15, 10.20), respectively. No significant difference was found between dulaglutide versus placebo. Compared with insulin, exe_lar (OR 1.92 (95%CI: 1.27, 2.92)) and liraglutide (OR 1.41 (95%CI: 1.03, 1.93)) were associated with higher incidence of HbA1c <7%. The incidence of HbA1c<7% was also increased when liraglutide versus sitagliptin (OR 1.91 (95%CI: 1.22, 3.00)). Besides, exenatide decreased the incidence of HbA1c <7% in comparison with exe_lar by 0.51 (95%CI: 0.33, 0.77). No statistically significant difference was found between other GLP-1 RAs versus placebo or active comparators in their effects on HbA1c <7%.

Regarding HbA1c <6.5%, exenatide, liraglutide, lixisenatide and taspoglutide were significantly increased the incidence in comparison with placebo by 3.14 (95% CI: 1.97, 5.01), 5.57 (95%CI: 2.75, 11.25), 3.36 (95%CI: 2.51, 4.50) and 6.89 (95%CI: 4.93, 9.62), respectively. No significant difference was found between albiglutide, dulaglutide or exe_lar versus placebo. Besides, compared with insulin, exe_lar (OR 3.55 (95%CI: 1.53, 8.23)) was associated with higher incidence of HbA1c<6.5%. Exenatide could also increase the incidence of HbA1c<6.5% when compared with exe_lar (OR 0.40 (95%CI: 0.22, 0.73)). No statistically significant difference was found between GLP-1 RAs and other active comparators in their effects on HbA1c <6.5%. ORs with 95%CIs were listed in Fig 3D.

### Network meta-analysis of individual GLP-1 RAs

**Fig 4:**
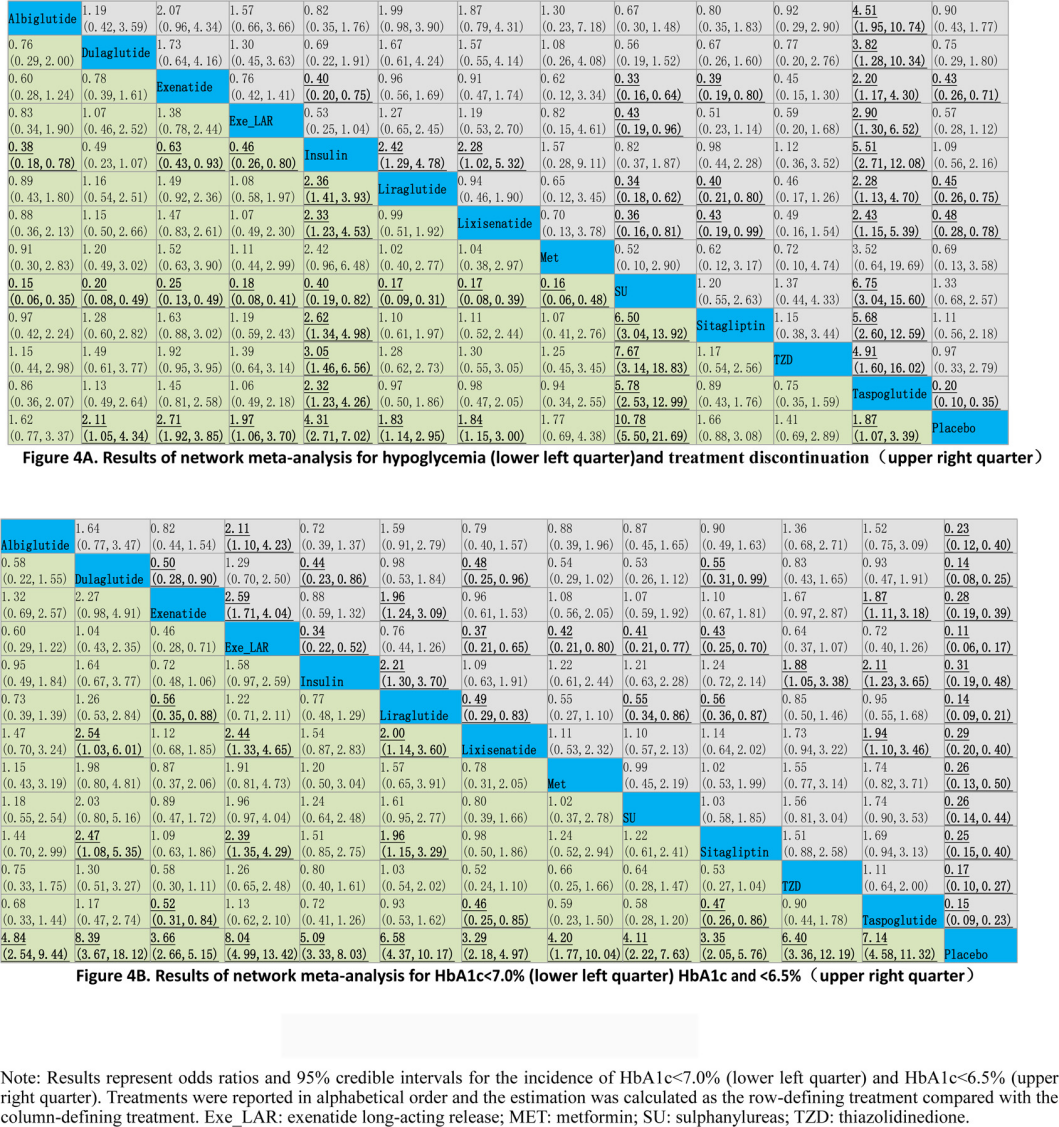
Results of network meta-analysis. (A) Results of network meta-analysis for hypoglycemia (lower left quarter)and treatment discontinuation(upper right quarter). (B) Results of network meta-analysis for HbA1c<7.0% (lower left quarter) HbA1c and <6.5%(upper right quarter).

Results of network meta-analysis among GLP-1 RAs, placebo and active comparators were displayed in Fig 4. As shown in Fig 4A, all GLP-1 RAs except for albiglutide increased the risk of hypoglycemia with range from 1.83 (95%CrI: 1.14, 2.95) to 2.71 (95%CrI: 1.92, 3. 85) when compared with placebo. Compared with insulin, all GLP-1 RAs except for dulaglutide reduced the risk of hypoglycemia with range from 0.38 (95%CrI: 0.18, 0.78) to 0.63 (95%CrI: 0.43, 0.93). Similar findings were observed between all GLP-1 RAs and SU with ORs varied from 0.15 (95%CrI: 0.06, 0.35) to 0.25 (95%CrI: 0.13, 0.49). No statistically significant difference was found between GLP-1 RAs versus Met, sitagliptin or TZD.

Regarding treatment discontinuation, exenatide, liraglutide, lixisenatide and taspoglutide significantly increase the incidence when compared with placebo (range of ORs: 2.08 (95%CrI: 1.28, 3.57) to 5.00 (95%CrI: 2.86, 10.00)) or insulin (range of ORs: 2.28 (95%CrI: 1.02, 5.32) to 5.51 (95%CrI: 2.71, 12.08)). Similar associations were found when exenatide, exe_lar, liraglutide, lixisenatide and taspoglutide were compared to SU (range of ORs: 2.33 (95%CrI: 1.04, 5.26) to 6.75 (95%CrI: 3.04, 15.60)). Exenatide, liraglutide, lixisenatide and taspoglutide were associated with higher incidence of treatment discontinuation with range from 2.33 (95%CrI: 1.01, 5.26) to 5.68 (95%CrI: 2.60, 12.59) when compared with sitagliptin. Increase was found between taspoglutide and TZD (OR 4.91 (95%CrI: 1.60, 16.02)) in their effects on the incidence of treatment discontinuation.

Fig 4B displayed the effect on HbA1c <7% and HbA1c <6.5%. For HbA1c <7%, all GLP-1 RAs significantly increased the incidence with range from 3.29 (95%CrI: 2.18, 4.97) to 8.39 (95%CrI: 3.67, 18.12) versus placebo. Dulaglutide, exe_lar, liraglutide and taspoglutide were associated with higher incidence with the range from 1.96 (95%CrI: 1.15, 3.29) to 2.47 (95%CrI: 1.08, 5.35) when compared with sitagliptin. No statistically significant difference was found between GLP-1 RAs versus insulin, Met, SU or TZD.

Regarding HbA1c <6.5%, all GLP-1 RAs significantly increased the incidence with range from 3.45 (95%CrI: 2.50, 5.00) to 9.09 (95%CrI: 5.88, 16.67) in comparison to placebo. Dulaglutide, exe_lar, liraglutide and taspoglutide were associated with higher incidence with the range from 2.11 (95%CrI: 1.23, 3.65) to 2.94 (95%CrI: 1.92, 4.55) when compared with insulin. Significant increase incidence of HbA1c <6.5% was found between exe_lar and Met (OR 2.38 (95%CrI: 1.25, 4.76). In comparison to SU, exe_lar and liraglutide significantly increase the incidence of HbA1c <6.5% by 2.44 (95%CrI: 1.25, 4.76) and 1.82 (95%CrI: 1.16, 2.94), respectively. Besides, significantly increase incidence was found between dulaglutide, exe_lar, liraglutide versus sitagliptin with range from 1.79 (95%CrI: 1.15, 2.78) to 2.33 (95%CrI: 1.43, 4.00). No statistically significant difference was found between GLP-1 RAs and TZD in their effects on HbA1c <6.5%.

### Ranking of 13 treatments on hypoglycemia, treatment discontinuation, HbA1c<7.0% and HbA1c<6.5%

Table 2 showed the mean values of SUCRA (S1 Fig) for providing the hierarchy of 13 treatments on hypoglycemia, treatment discontinuation, HbA1c<7.0% and HbA1c<6.5% based on the absolute rank probabilities. According to SUCRAs, placebo, TZD and albiglutide had the best decrease effect on hypoglycemia, with probability of 4.08%, 24.95% and 35.19% respectively. SU, sitagliptin and insulin had the lowest probability of treatment discontinuation with rates of 13.44%, 23.01% and 23.63%, respectively. With respect to HbA1c<7.0% and HbA1c<6.5%, exe_lar and dulaglutide lowering glycemic level most among 13 treatments.

**Table 2:** Ranking: probability from SUCRA of efficacy and acceptability of glycemic control of different GLP-1s.

CODE	Treatments	Hypoglycemia		Treatment discontinuation		HbA1c <7.0%		HbA1c <6.5%	
		SUCRA	Rank	SUCRA	Rank	SUCRA	Rank	SUCRA	Rank
1	Albiglutide	**0.3519**	**3**	0.3644	6	0.4989	7	0.4805	6
2	Dulaglutide	0.5555	10	0.4941	7	**0.8460**	**2**	**0.8186**	**2**
3	Exe_LAR	0.5027	9	0.6290	9	**0.8718**	**1**	**0.9531**	**1**
4	Exenatide	0.7519	11	0.7903	12	0.2802	10	0.3122	10
5	Insulin	0.9090	12	**0.2363**	**3**	0.5381	6	0.2145	12
6	Liraglutide	0.4444	6	0.7657	11	0.7421	4	**0.8149**	**3**
7	Lixisenatide	0.4461	7	0.7311	10	0.2239	12	0.2901	11
8	Met	0.4224	5	0.5147	8	0.4067	8	0.3779	8
9	Sitagliptin	0.3668	4	**0.2301**	**2**	0.2362	11	0.3905	7
10	SU	0.9994	13	**0.1344**	**1**	0.3690	9	0.3652	9
11	Taspoglutide	0.4597	8	0.9901	13	**0.7859**	**3**	0.7792	4
12	TZD	**0.2495**	**2**	0.3226	5	0.7011	5	0.7033	5
13	Placebo	**0.0408**	**1**	0.2972	4	0.0001	13	0.0000	13

Note: Rank: probability of being the best treatment, of being the second best, the third best and so on, among the 13 comparisons. SUCRA: surface under the cumulative ranking curve. As for hypoglycemia and treatment discontinuation first one means the best safety. As for HbA1c<7.0% and HbA1c<6.5% first one means has the best efficacy.

### Model fit and inconsistence check

Statistical inconsistency between direct and indirect comparisons was generally low for four outcomes. Most loops (networks of three or four comparisons that arise when collating studies involving different selections of competing treatments) were consistent, since their 95% CIs included 0 according to the forest plots, which meant the direct estimation of the summary effect did not differentiate from the indirect estimation. The summary estimations of network meta-analysis are relatively robust.

The model fit was evaluated using the posterior mean of the residual deviance ${\bar{\text{D}}}_{\text{res}}$. The values of the ${\bar{\text{D}}}_{\text{res}}$ for hypoglycemia, treatment discontinuation, HbA1c<7.0% and HbA1c<6.5% were 121.98, 83.34, 131.68 and 86.80 respectively, which were close to corresponding 152, 104,145 and 104 of the number of data points for four outcomes, meaning that model’s overall fit is relatively satisfactory.

## Discussion

Aside from adequate glycemic control, increasing attention is being paid to the hypoglycemia and treatment discontinuation effect of GLP-1 RAs recently [14, 15]. Our network meta-analysis suggested that all GLP-1 RAs significantly increase the risk of hypoglycemia compared with placebo (except for albiglutide), and reduce the risk of hypoglycemia compared with insulin (except for dulaglutide) and SU. In terms of the increasing incidence of treatment discontinuation, exenatide, liraglutide, lixisenatide and taspoglutide had significant effect when compared with either placebo, insulin, SU or sitagliptin, and exe_lar only increased the incidence of treatment discontinuation significantly when compared with SU. This was accompanied by taspoglutide in comparison to TZD. Besides, all GLP-1 RAs decreased glycemic level compared with placebo, and dulaglutide, exe_lar, liraglutide and taspoglutide had significant lowering effect when compared with sitagliptin (HbA1c<7.0%) and insulin(HbA1c<6.5%). Regarding to HbA1c <6.5%, there was also a significant lowing effect for exe_lar and liraglutide in comparison to SU and sitagliptin, dulaglutide in comparison to sitagliptin, exe_lar in comparison to Met.

### Effect on hypoglycemia

Hypoglycemia is a common complication of intensive diabetes therapy, which could cause fall, seizure, coma, and even death[109]. The UK Prospective Diabetes Study (UKPDS) reported that maintenance of tight glycemic control in T2DM with insulin treated led to a significant increase in the incidence of hypoglycemia[110]. Our network meta-analysis showed that the significant increasing in the incidence of hypoglycemia was associated with all GLP-1 RAs except for albiglutide, which was consistent with Riddle’s study [90]. Riddle’s results showed that the incidence of symptomatic hypoglycemia was 28% for lixisenatide and 22% for placebo, and 1.2% subjects had severe hypoglycemia with lixisenatide vs. 0.0% with placebo. While, the beneficial hypoglycemia lowering effect of all GLP-1 RAs was observed when compared with insulin (except for dulaglutide) and SU, which was consistent with previous reviews[10, 111] and clinical trials[112]. Besides, studies also reported that the incidence of hypoglycemia was similar across GLP-1 RA treatment groups, and most of patients with hypoglycemia had the history of treating with concomitant SU therapy [111, 113].

To date, the mechanism of hypoglycemia for T2DM has not been clearly identified. It may involve complex regulation, but it has been shown that β-cell failure precede defects of α-cell response to lowering glucagon levels in T2DM, indicating that the counter-regulatory effect of glucagon to hypoglycemia is impaired in T2DM[114, 115]. Fukuda’s[116] study reported that the degree of α-cell dysfunction is related with the lack of β-cell function in diabetes. Commonly, insulin represses glucagon secretion as a pulsatile manner, but this coordination is disrupted in patients with T2DM and it could potentially contribute to glucagon dysregulation[117]. So finally, the defect of an increment in glucagon secretion during hypoglycemia is the result of β-cell failure in advanced T2DM[118].

### Treatment discontinuation increasing effect

Our network meta-analysis showed that exenatide, liraglutide, lixisenatide and taspoglutide had significant increasing effect on the incidence of treatment discontinuation when compared with either placebo, insulin, SU or sitagliptin. Exe_lar only increased the incidence of treatment discontinuation when compared with SU. This was accompanied by taspoglutide in comparison to TZD. Similar results were indicated in relevant clinical trials[113].

Several reasons may be account for this. Firstly, All GLP-1 RAs are injected subcutaneously, and cannot be administered orally. The incidence of treatment discontinuation among patients who had injection site adverse events was high[119]. Secondly, the adverse events of GLP-1 RAs like nausea, diarrhea, and vomiting, also account for the incidence of treatment discontinuation[120]. Especially for the most commonly occurred nausea, which usually lasts a long time, is a tough experience for T2DM to bear.

### Glycemic level lowering effect

The beneficial glycemic level lowering effect of all GLP-1 RAs in our analysis was consistent with previous studies [10, 121]. Scheen’s [122] study reported that the HbA1c lowering potential for GLP-1 RAs is approximately at 1%–1.5% on average. A review of 8 head-to-head phase III clinical programs showed that the primary efficacy endpoint in all of the GLP-1 RAs was change in HbA1c from baseline with a noninferiority margin of 0.4%[111]. Similar results were indicated in relevant clinical trials. The significant glycemic level lowering effect of EXQW was observed in series of DURATION trials, with mean reductions of -0.9% to -1.63% [18, 44, 123–126]. Liraglutide was found to lower HbA1c by -0.9 to -1.1% [127].

Besides, our study also found that dulaglutide, exe_lar, liraglutide and taspoglutide had significant lowering effect when compared with sitagliptin (HbA1c<7.0%) and insulin(HbA1c<6.5%), which was consistent with Nauck’s results, which showed that dulaglutide 0.75 mg reducing HbA1c by 0.87%±0.06% versus sitagliptin reducing HbA1c by 0.39%±0.06% (P,0.001)[128]. Regarding to HbA1c <6.5%, our study also demonstrated that there was a significant lowering effect for exe_lar and liraglutide in comparison to SU and sitagliptin, dulaglutide in comparison to sitagliptin, exe_lar in comparison to Met.

### Strengths

A major strength of our study is the inclusion of a substantially greater number of trials of GLP-1 RAs than earlier meta-analysis[16, 20, 21], thus it is the largest completed evaluation of GLP-1 RAs’ effect on hypoglycemia, treatment discontinuation and glycemic level to date. Furthermore, the network meta-analysis based on Bayesian model makes indirect comparison among multiple treatments available, especially when there are few trials for direct comparison between different anti-diabetic drugs, such as comparisons between dulaglutide and insulin in our study. Network meta-analysis has been proved to be the most appropriate method for multiple treatments comparison to date [26, 129]. In addition, the network technique enables the estimation of the probability that one intervention is the best for one outcome. Thus it can provide an explicit ranking when many treatments are competing for one outcome. Our study provided the ranks of GLP-1 RAs and traditional anti-diabetic drugs on hypoglycemia, treatment discontinuation and glycemic level for the first time.

### Limitations

Several limitations are worthy to be mentioned. First, only trials only in English were included, and our literature search was from inception to June 1st, 2014, and didn’t include literatures published after June 1st, 2014, which may lead to potential publication bias and selection bias. Secondly, none of the trials included was specially designed to evaluate the effect of GLP-1 RAs on hypoglycemia, treatment discontinuation and glycemic level. Thirdly, the different duration of years of T2DM in 78 trials may cause heterogeneous, which may influence the different response to therapy and increase the possibility of hypoglycemia. Thus the results of our study should be considered as hypothesis generation, and any conclusions should be drawn with caution.

## Conclusion

In conclusion, our network meta-analysis presents the associations amongGLP-1 RAs, traditional anti-diabetic drugs and placebo on hypoglycemia, treatment discontinuation and glycemic level. GLP-1 RAs had the lowering effect on glycemic level, increasing effect on hypoglycemia and treatment discontinuation. While, GLP-1 RAs were associated with lower incidence of hypoglycemia when compared with active comparators. However, further evidence is necessary for more conclusive inferences on mechanisms underlying the increasing in hypoglycemia.

## Supporting Information

S1 Fig Plots for ranking probability of different dosing of GLP-1s on impact of SBP, DBP, heart rate and hypertension. (PDF)

S1 File PRISMA 2009 Checklist. (PDF)

S1 Table Quality of included trials by adjusted Jadad scale. (PDF)
