# Supplementary material for: Efficacy and Acceptability of Glycemic Control of Glucagon-Like Peptide-1 Receptor Agonists among Type 2 Diabetes: A Systematic Review and Network Meta-Analysis
Source: PLoS One. 2016 May 9;11(5):e0154206. doi: 10.1371/journal.pone.0154206 (PMC4861281; doi:10.1371/journal.pone.0154206)
Supplement: S1 Fig — (PDF) [file pone.0154206.s001.pdf]

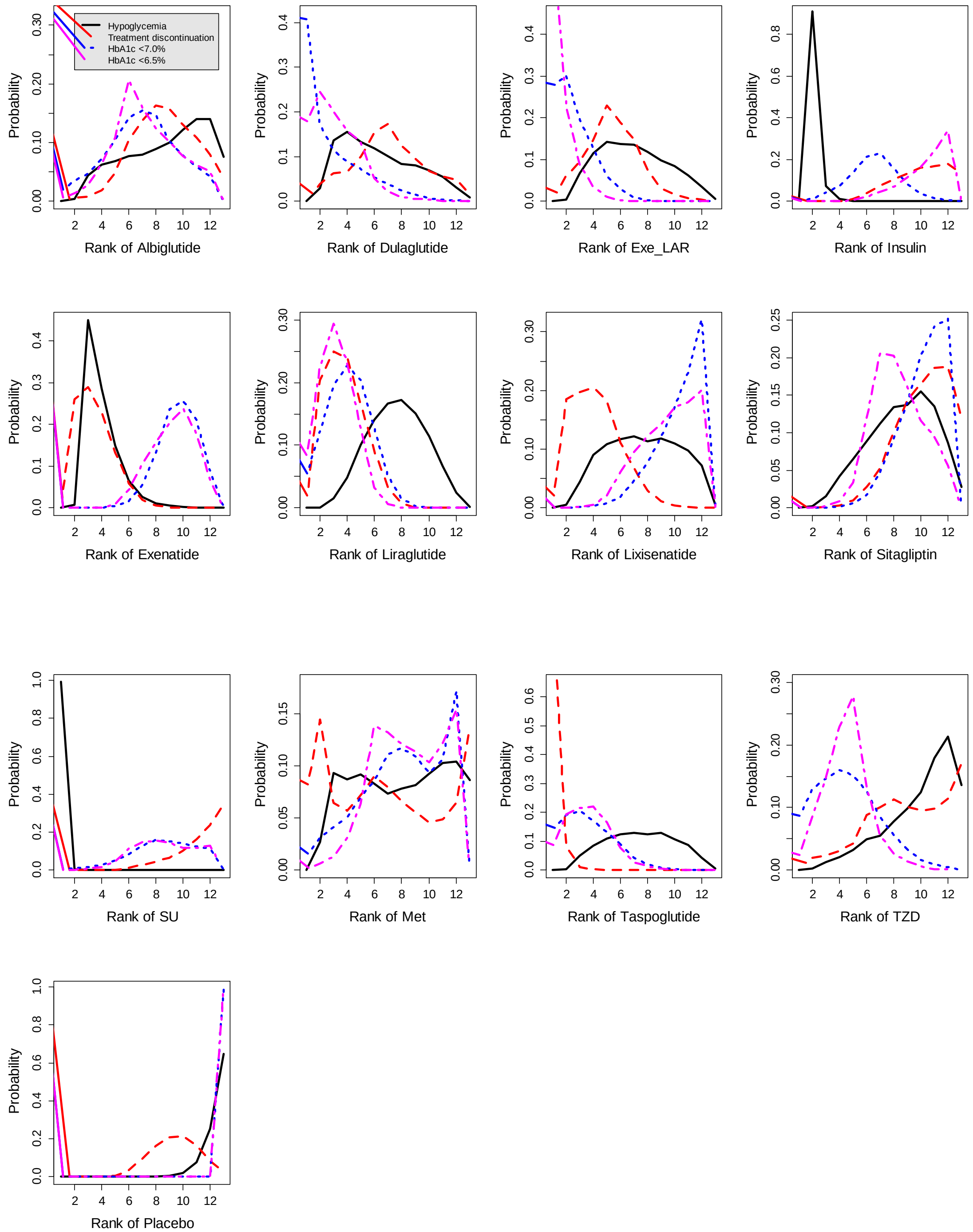

A. Plots of these rank probability (Rankograms)

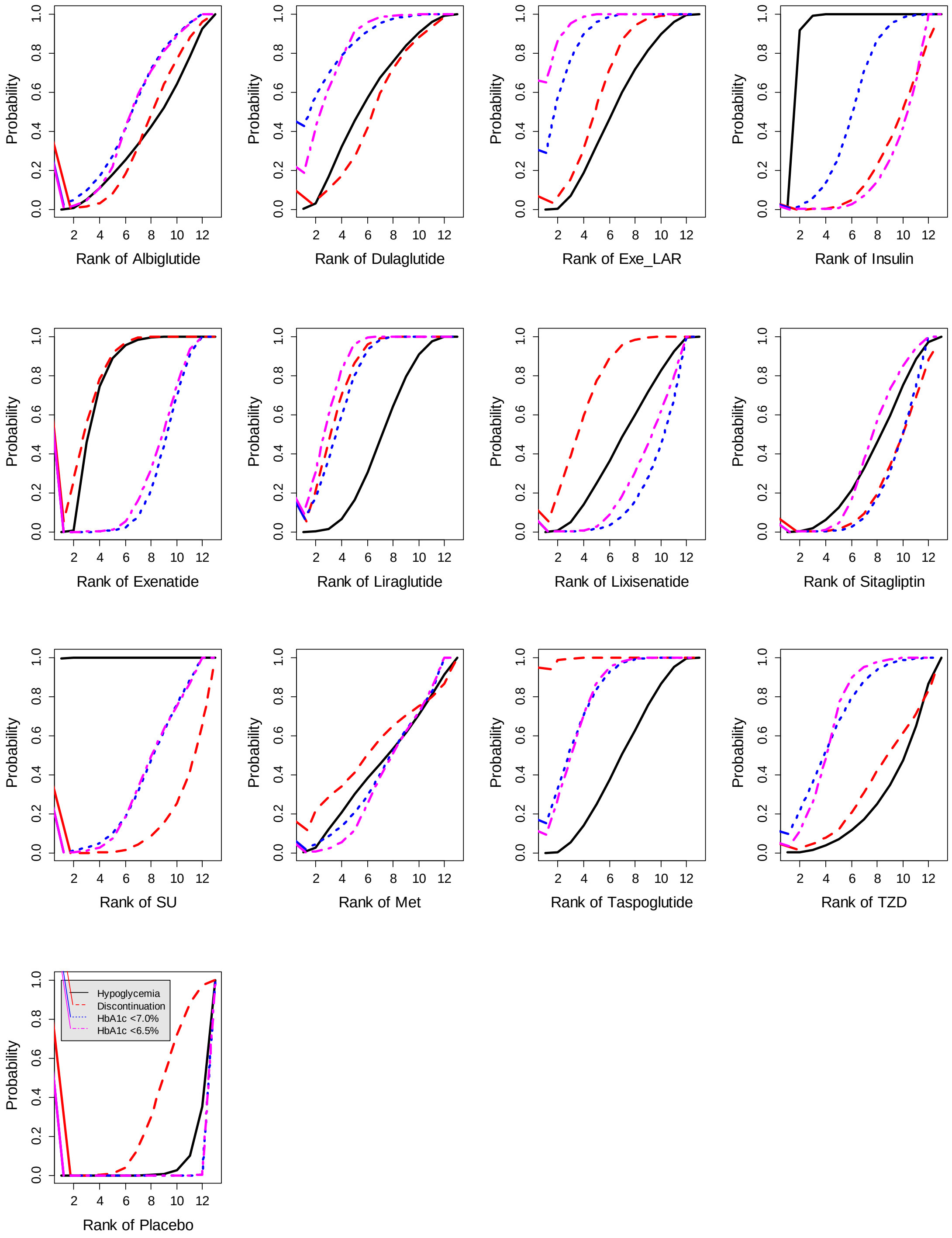

B. Plots of cumulative ranking probability (SUCRA)

Appendix figure1. Plots for ranking probability of of different dosing of GLP-1s on impact of SBP, DBP, heart rate and hypertension.

Note: SU: sulphanylureas; TZD:thiazolidinedione. Ranking: probability of being the best treatment, of being the second best, the third best and so on, among the 12 comparisons. SUCRA:surface under the cumulative ranking curve.
