# Supplementary material for: Efficacy and Acceptability of Glycemic Control of Glucagon-Like Peptide-1 Receptor Agonists among Type 2 Diabetes: A Systematic Review and Network Meta-Analysis
Source: PLoS One. 2016 May 9;11(5):e0154206. doi: 10.1371/journal.pone.0154206 (PMC4861281; doi:10.1371/journal.pone.0154206)
Supplement: S1 File — (PDF) [file pone.0154206.s002.pdf]

PRISMA 2009 Checklist

Section/topic	#	Checklist item	Reported on page #
**TITLE**			
Title	1	Identify the report as a systematic review, meta-analysis, or both.	Page 1 Line 1-3
**ABSTRACT**			
Structured summary	2	Provide a structured summary including, as applicable: background; objectives; data sources; study eligibility criteria, participants, and interventions; study appraisal and synthesis methods; results; limitations; conclusions and implications of key findings; systematic review registration number.	Page 2
**INTRODUCTION**			
Rationale	3	Describe the rationale for the review in the context of what is already known.	Page 3 Line 68-95
Objectives	4	Provide an explicit statement of questions being addressed with reference to participants, interventions, comparisons, outcomes, and study design (PICOS).	Page 3 Line 96-103
**METHODS**			
Protocol and registration	5	Indicate if a review protocol exists, if and where it can be accessed (e.g., Web address), and, if available, provide registration information including registration number.	Page 3 Line 105
Eligibility criteria	6	Specify study characteristics (e.g., PICOS, length of follow-up) and report characteristics (e.g., years considered, language, publication status) used as criteria for eligibility, giving rationale.	Page 4 Line 121-126
Information sources	7	Describe all information sources (e.g., databases with dates of coverage, contact with study authors to identify additional studies) in the search and date last searched.	Page 4 Line 107-109; 116-120
Search	8	Present full electronic search strategy for at least one database, including any limits used, such that it could be repeated.	Page 4 Line 110-115
Study selection	9	State the process for selecting studies (i.e., screening, eligibility, included in systematic review, and, if applicable, included in the meta-analysis).	Page 4 Line 126-127

Data collection process	10	Describe method of data extraction from reports (e.g., piloted forms, independently, in duplicate) and any processes for obtaining and confirming data from investigators.	Page 4 Line 129-136
Data items	11	List and define all variables for which data were sought (e.g., PICOS, funding sources) and any assumptions and simplifications made.	Page 4 Line 129-134
Risk of bias in individual studies	12	Describe methods used for assessing risk of bias of individual studies (including specification of whether this was done at the study or outcome level), and how this information is to be used in any data synthesis.	Page 4 Line 137-140
Summary measures	13	State the principal summary measures (e.g., risk ratio, difference in means).	Page 4 Line 141-145; Page 5 Line 157-159; 162-168
Synthesis of results	14	Describe the methods of handling data and combining results of studies, if done, including measures of consistency (e.g., I^2) for each meta-analysis.	Page 4 Line 141-145; Page 5 146-173

Section/topic	#	Checklist item	Reported on page #
Risk of bias across studies	15	Specify any assessment of risk of bias that may affect the cumulative evidence (e.g., publication bias, selective reporting within studies).	Page 5 Line 174-182
Additional analyses	16	Describe methods of additional analyses (e.g., sensitivity or subgroup analyses, meta-regression), if done, indicating which were pre-specified.	NA
**RESULTS**			
Study selection	17	Give numbers of studies screened, assessed for eligibility, and included in the review, with reasons for exclusions at each stage, ideally with a flow diagram.	Page 6 Line 188-189;

PRISMA 2009 Checklist

			Figure 1
Study characteristics	18	For each study, present characteristics for which data were extracted (e.g., study size, PICOS, follow-up period) and provide the citations.	Page 6 Line 190-204; Page 7,8;Page 9 Line 205-207
Risk of bias within studies	19	Present data on risk of bias of each study and, if available, any outcome level assessment (see item 12).	Page 9 Line 209-214
Results of individual studies	20	For all outcomes considered (benefits or harms), present, for each study: (a) simple summary data for each intervention group (b) effect estimates and confidence intervals, ideally with a forest plot.	Page 9 Line 215-226
Synthesis of results	21	Present results of each meta-analysis done, including confidence intervals and measures of consistency.	Page 9 Line 227-244; Page 10; Page 11 Line 286-307
Risk of bias across studies	22	Present results of any assessment of risk of bias across studies (see Item 15).	Page 12 Line 325-337
Additional analysis	23	Give results of additional analyses, if done (e.g., sensitivity or subgroup analyses, meta-regression [see Item 16]).	Page 11 Line 308-320; Page 12 Line 321-324
**DISCUSSION**			
Summary of evidence	24	Summarize the main findings including the strength of evidence for each main outcome; consider their relevance to key groups (e.g., healthcare providers, users, and policy makers).	Page 12 Line

			339-352; Page 13; Page 14 Line 393-417
Limitations	25	Discuss limitations at study and outcome level (e.g., risk of bias), and at review-level (e.g., incomplete retrieval of identified research, reporting bias).	Page 14 Line 433; Page 15 Line 434-438
Conclusions	26	Provide a general interpretation of the results in the context of other evidence, and implications for future research.	Page 15 Line 439-447
**FUNDING**			
Funding	27	Describe sources of funding for the systematic review and other support (e.g., supply of data); role of funders for the systematic review.	Page 15 Line 454-458

From: Moher D, Liberati A, Tetzlaff J, Altman DG, The PRISMA Group (2009). Preferred Reporting Items for Systematic Reviews and Meta-Analyses: The PRISMA Statement. PLoS Med 6(6): e1000097. doi:10.1371/journal.pmed1000097

For more information, visit: **www.prisma-statement.org**.
