# Supplementary material for: Efficacy and Acceptability of Glycemic Control of Glucagon-Like Peptide-1 Receptor Agonists among Type 2 Diabetes: A Systematic Review and Network Meta-Analysis
Source: PLoS One. 2016 May 9;11(5):e0154206. doi: 10.1371/journal.pone.0154206 (PMC4861281; doi:10.1371/journal.pone.0154206)
Supplement: S1 Table — (PDF) [file pone.0154206.s003.pdf]

Appendix table 1. Quality of included trials by adjusted Jadad scale

No	Study ID	Clinical trial code	Adequate sequence generation	Allocation concealment	Blinding of participants and personnel	Dropout addressed	ITT
1	Ahren B, 2013[1]	NCT00712673	Adequate	Adequate	Adequate, DB	Adequate	Y
2	Ahren B, 2014[2]	NCT00838903	Adequate	Adequate	Adequate, DB	Adequate	Y
3	Apovian CM, 2010[3]	NCT00375492	Adequate	Adequate	Adequate, DB	Adequate	Y
4	Barnett AH, 2007[4]	NCT00099619	Adequate	Adequate	Open Label	Adequate	Y
5	Bergenstal R, 2009[5]	NCT00097877	Adequate	Adequate	Open Label	Adequate	Y
6	Bergenstal RM, 2010[6]	NCT00637273	Adequate	Adequate	Adequate, DB	Adequate	Y
7	Bergenstal RM, 2012[7]	NCT00754988	Adequate	Unclear	Unclear, DB	Adequate	N
8	Blevins T, 2011[8]	NCT00877890	Adequate	Adequate	Open Label	Adequate	Y
9	Bolli G, 2013[9]	NCT00763451	Adequate	Unclear	Unclear, DB	Unclear	N
10	Bunck MC, 2009[10]	NCT00097500	Adequate	Unclear	Open Label	Adequate	Y
11	Buse JB, 2004[11]	NCT00039026	Adequate	Unclear	Adequate, DB	Adequate	Y
12	Buse JB, 2009[12]	NCT00518882	Unclear	Adequate	Open Label	Adequate	Y
13	Buse JB, 2011[13]	NCT00765817	Adequate	Adequate	Adequate, DB	Adequate	Y
14	Buse JB, 2013[14]	NCT01029886	Adequate	Adequate	Open Label	Adequate	Y
15	Charbonnel B, 2013[15]	NCT01296412	Adequate	Unclear	Open Label	Adequate	Y
16	Davies M, 2013[16]	NCT01003184	Adequate	Adequate	Open Label	Adequate	Y
17	Davies MJ, 2009[17]	NCT00360334	Adequate	Unclear	Open Label	Adequate	Y
18	Davis SN, 2007[18]	NCT00099333	Unclear	Unclear	Open Label	Adequate	Y
19	DeFronzo RA, 2005[19]	NCT00039013	Unclear	Unclear	Adequate, TB	Adequate	Y
20	DeFronzo RA, 2010[20]	NCT00135330	Adequate	Unclear	Open Label	Adequate	Y

21	Derosa G, 2012[21]	NR	Adequate	Adequate	Adequate, DB	Adequate	Y
22	Diamant M, 2012[22]	NCT00641056	Adequate	Adequate	Open Label	Adequate	Y
23	Drucker DJ, 2008[23]	NCT00308139	Unclear	Unclear	Open Label	Adequate	Y
24	Fonseca VA, 2012[24]	NCT00688701	Adequate	Adequate	Adequate, DB	Adequate	Y
25	Gallwitz B, 2011[25]	NCT00434954	Adequate	Unclear	Open Label	Unclear	Y
26	Gallwitz B, 2012[26]	NCT00359762	Adequate	Unclear	Open Label	Unadequate	Y
27	Gao Y, 2009[27]	NCT00324363	Adequate	Adequate	Adequate, DB	Adequate	Y
28	Garder A, 2011[28]	NCT00294723	Adequate	Adequate	Open Label	Adequate	Y
29	Heine RJ, 2005[29]	NCT00082381	Adequate	Adequate	Open Label	Adequate	Y
30	Henry RR, 2012[30]	NCT00744367	Adequate	Adequate	Adequate, DB	Adequate	Y
31	Hollander P, 2013[31]	NCT00823992	Adequate	Adequate	Adequate, DB	Adequate	Y
32	Inagaki N, 2012[32]	NCT00935532	Adequate	Unclear	Open Label	Adequate	N
33	Iwamoto K, 2009[33]	NCT00612794	Adequate	Adequate	Adequate, DB	Unclear	Y
34	Ji LN, 2013[34]	NCT00917267	Adequate	Unclear	Open Label	Adequate	Y
35	Kadowaki T, 2009[35]	NCT00382239	Unclear	Unclear	Adequate, DB	Adequate	Y
36	Kadowaki T, 2011[36]	NCT00577824	Adequate	Unclear	Adequate, DB	Adequate	Y
37	Kendall DM, 2005[37]	NCT00035984	Unclear	Unclear	Adequate, DB	Adequate	Y
38	Kim D, 2007[38]	NCT00103935	Adequate	Adequate	Adequate, DB	Adequate	Y
39	Li CJ, 2012[39]	NR	Adequate	Adequate	Adequate, DB	Adequate	N
40	Liutkus J, 2010[40]	NCT00603239	Adequate	Adequate	Adequate, DB	Adequate	Y
41	Marre M, 2009[41]	NCT00318422	Unclear	Unclear	Adequate, DB	Adequate	Y
42	Mathieu C, 2014[42]	NCT01388361	Adequate	Unclear	Open Label	Adequate	Y
43	Moretto TJ, 2008[43]	NCT00381342	Adequate	Adequate	Adequate, DB	Adequate	Y
44	Nauck M, 2014[44]	NCT00734474	Adequate	Adequate	Adequate, DB	Adequate	Y

45	Nauck MA, 2007[45]	NCT00082407	Adequate	Adequate	Open Label	Adequate	Y
46	Nauck MA, 2009[46]	NCT00423501	Adequate	Adequate	Adequate, DB	Adequate	Y
47	Nauck M, 2009[47]	NCT00318461	Adequate	Adequate	Adequate, DB	Adequate	Y
48	Nauk M, 2013[48]	NCT00755287	Adequate	Adequate	Open Label	Adequate	Y
49	NCT00620282, 2011[49]	NCT00620282	Adequate	Unclear	Unclear, DB	Unclear	N
50	NCT00667732, 2013[50]	NCT00667732	Adequate	Adequate	Adequate, DB	Adequate	N
51	NCT00701935, 2013[51]	NCT00701935	unclear	unclear	Adequate, DB	unclear	Y
52	Pinget M, 2013[52]	NCT00763815	Adequate	Adequate	Adequate, DB	Adequate	Y
53	Pratley R, 2011[53]	NCT00700817	Adequate	Adequate	Open Label	Adequate	N
54	Pratley RE , 2013[54]	NCT00909597	Adequate	Adequate	Adequate, DB	Adequate	Y
55	Pratley RE, 2014[55]	NCT01128894	Adequate	Adequate	Open Label	Adequate	Y
56	Ratner R, 2010[56]	NCT00460941	Adequate	Unclear	Adequate, DB	Adequate	Y
57	Ratner RE, 2010[57]	NR	Adequate	Adequate	Adequate, DB	Adequate	Y
58	Raz I , 2012[58]	NCT00744926	Adequate	Adequate	Adequate, DB	Adequate	Y
59	Riddle MC,2013[59]	NCT00975286	Adequate	Adequate	Adequate, DB	Adequate	Y
60	Riddle MC，2013[60]	NCT00715624	Adequate	Adequate	Adequate, DB	Adequate	Y
61	Rosenstock J, 2013[61]	NCT00717457	Adequate	Unclear	Open Label	Adequate	Y
62	Rosenstock J, 2014[62]	NCT00713830	Adequate	Unclear	Unclear, DB	Adequate	Y
63	Rosenstock J, 2014[63]	NCT00976391	Adequate	Unclear	Open Label	Adequate	Y
64	Rosenstock J, 2009[64]	NCT00518115	Unclear	Unclear	Adequate, DB	Unclear	Y
65	Rosenstock J, 2013[65]	NCT00707031	Adequate	Unclear	Open Label	Adequate	Y
66	Russell-Jones D, 2009[66]	NCT00331851	Adequate	Adequate	Adequate, DB	Adequate	Y
67	Russell-Jones D,	NCT00676338	Adequate	Adequate	Adequate, DB	Adequate	Y

	2012[67]						
68	Seino Y, 2008[68]	NCT00154414	Adequate	Adequate	Adequate, DB	Adequate	Y
69	Seino Y, 2010[69]	NCT00393718	Unclear	Unclear	Unclear	Unclear	Y
70	Seino Y, 2012[70]	NCT00866658	Adequate	Adequate	Adequate, DB	Adequate	Y
71	Seino Y, 2014[71]	NCT01098461	Adequate	Adequate	Adequate, DB	Adequate	N
72	Umpierrez G, 2014[72]	NCT01126580	Adequate	Adequate	Adequate, DB	Adequate	Y
73	Umpierrez GE, 2011[73]	NCT00630825	Adequate	Unclear	Adequate, DB	Adequate	Y
74	Wysham C, 2014[74]	NCT01064687	Adequate	Adequate	Adequate, DB	Adequate	Y
75	Yang W, 2011[75]	NCT00614120	Unclear	Unclear	Adequate, DB	Adequate	N
76	Yuan GH, 2012[76]	NR	Adequate	Unclear	Adequate, DB	Adequate	Y
77	Zinman B, 2007[77]	NCT00099320	Adequate	Adequate	Adequate, DB	Adequate	Y
78	Zinman B, 2009[78]	NCT00333151	Adequate	Adequate	Adequate, DB	Adequate	Y

Note: DB: double blinding; TB: triple blinding; ITT, intention-to-treat analysis; NR: not reported

References

[1] AHREN B, LEGUIZAMO DIMAS A, MIOSSEC P, et al. Efficacy and Safety of Lixisenatide Once-Daily Morning or Evening Injections in Type 2 Diabetes Inadequately Controlled on Metformin (GetGoal-M) [J]. Diabetes care, 2013, 36(9): 2543-50.

[2] AHREN B, JOHNSON S L, STEWART M, et al. HARMONY 3: 104-Week Randomized, Double-Blind, Placebo- and Active-Controlled Trial Assessing the Efficacy and Safety of Albiglutide Compared With Placebo, Sitagliptin, and Glimepiride in Patients With Type 2 Diabetes Taking Metformin [J]. Diabetes care, 2014,

[3] APOVIAN C M, BERGENSTAL R M, CUDDIHY R M, et al. Effects of Exenatide Combined with Lifestyle Modification in Patients with Type 2 Diabetes [J]. American Journal of Medicine, 2010, 123(5): 468.e9-.e17.

[4] BARNETT A H, BURGER J, JOHNS D, et al. Tolerability and efficacy of exenatide and titrated insulin glargine in adult patients with type 2 diabetes previously uncontrolled with metformin or a sulfonylurea: A multinational, randomized, open-label, two-period, crossover noninferiority trial [J]. Clinical Therapeutics, 2007, 29(11): 2333-48.

[5] BERGENSTAL R, LEWIN A, BAILEY T, et al. Efficacy and safety of biphasic insulin aspart 70/30 versus exenatide in subjects with type 2 diabetes failing to achieve glycemic control with metformin and a sulfonylurea [J]. Curr Med Res Opin, 2009, 25(1): 65-75.

[6] BERGENSTAL R M, WYSHAM C, MACCONELL L, et al. Efficacy and safety of exenatide once weekly versus sitagliptin or pioglitazone as an adjunct to metformin for treatment of type 2 diabetes (DURATION-2): a randomised trial [J]. Lancet, 2010, 376(9739): 431-9.

[7] BERGENSTAL R M, FORTI A, CHIASSON J L, et al. Efficacy and safety of taspoglutide versus sitagliptin for type 2 diabetes mellitus (T-Emerge 4 Trial) [J]. Diabetes Therapy, 2012, 3(1): 1-19.

[8] BLEVINS T, PULLMAN J, MALLOY J, et al. DURATION-5: exenatide once weekly resulted in greater improvements in glycemic control compared with exenatide twice daily in patients with type 2 diabetes [J]. The Journal of clinical endocrinology and metabolism, 2011, 96(5): 1301-10.

[9] BOLLI G B, MUNTEANU M, DOTSENKO S, et al. Efficacy and safety of lixisenatide once daily vs. placebo in people with Type 2 diabetes insufficiently controlled on metformin (GetGoal-F1) [J]. Diabetic medicine : a journal of the British Diabetic Association, 2013,

[10] BUNCK M C, DIAMANT M, CORNER A, et al. One-year treatment with exenatide improves beta-cell function, compared with insulin glargine, in metformin-treated type 2 diabetic patients: a randomized, controlled trial [J]. Diabetes care, 2009, 32(5): 762-8.

[11] BUSE J B, HENRY R R, HAN J, et al. Effects of exenatide (exendin-4) on glycemic control over 30 weeks in sulfonylurea-treated patients with type 2 diabetes [J]. Diabetes care, 2004, 27(11): 2628-35.

[12] BUSE J B, ROSENSTOCK J, SESTI G, et al. Liraglutide once a day versus exenatide twice a day for type 2 diabetes: a 26-week randomised, parallel-group, multinational, open-label trial (LEAD-6) [J]. Lancet, 2009, 374(9683): 39-47.

[13] BUSE J B, BERGENSTAL R M, GLASS L C, et al. Use of twice-daily exenatide in Basal insulin-treated patients with type 2 diabetes: a randomized, controlled trial [J]. Ann Intern Med, 2011, 154(2): 103-12.

[14] BUSE J B, NAUCK M, FORST T, et al. Exenatide once weekly versus liraglutide once daily in patients with type 2 diabetes (DURATION-6): a randomised, open-label study [J]. Lancet, 2013,

381(9861): 117-24.
[15] CHARBONNEL B, STEINBERG H, EYMARD E, et al. Efficacy and safety over 26 weeks of an oral treatment strategy including sitagliptin compared with an injectable treatment strategy with liraglutide in patients with type 2 diabetes mellitus inadequately controlled on metformin: a randomised clinical trial [J]. Diabetologia, 2013, 56(7): 1503-11.
[16] DAVIES M, HELLER S, SREENAN S, et al. Once-weekly exenatide versus once- or twice-daily insulin detemir: randomized, open-label, clinical trial of efficacy and safety in patients with type 2 diabetes treated with metformin alone or in combination with sulfonylureas [J]. Diabetes care, 2013, 36(5): 1368-76.
[17] DAVIES M J, DONNELLY R, BARNETT A H, et al. Exenatide compared with long-acting insulin to achieve glycaemic control with minimal weight gain in patients with type 2 diabetes: Results of the helping evaluate exenatide in patients with diabetes compared with long-acting insulin (HEELA) study [J]. Diabetes, Obesity and Metabolism, 2009, 11(12): 1153-62.
[18] DAVIS S N, JOHNS D, MAGGS D, et al. Exploring the substitution of exenatide for insulin in patients with type 2 diabetes treated with insulin in combination with oral antidiabetes agents [J]. Diabetes care, 2007, 30(11): 2767-72.
[19] DEFRONZO R A, RATNER R E, HAN J, et al. Effects of exenatide (exendin-4) on glycemic control and weight over 30 weeks in metformin-treated patients with type 2 diabetes [J]. Diabetes care, 2005, 28(5): 1092-100.
[20] DEFRONZO R A, TRIPLITT C, QU Y, et al. Effects of exenatide plus rosiglitazone on (beta)-cell function and insulin sensitivity in subjects with type 2 diabetes on metformin [J]. Diabetes care, 2010, 33(5): 951-7.
[21] DEROSA G, FRANZETTI I G, QUERCI F, et al. Exenatide plus metformin compared with metformin alone on beta-cell function in patients with Type 2 diabetes [J]. Diabetic medicine : a journal of the British Diabetic Association, 2012, 29(12): 1515-23.
[22] DIAMANT M, VAN GAAL L, STRANKS S, et al. Safety and efficacy of once-weekly exenatide compared with insulin glargine titrated to target in patients with type 2 diabetes over 84 weeks [J]. Diabetes care, 2012, 35(4): 683-9.
[23] DRUCKER D J, BUSE J B, TAYLOR K, et al. Exenatide once weekly versus twice daily for the treatment of type 2 diabetes: a randomised, open-label, non-inferiority study [J]. Lancet, 2008, 372(9645): 1240-50.
[24] FONSECA V A, ALVARADO-RUIZ R, RACCAH D, et al. Efficacy and Safety of the Once-Daily GLP-1 Receptor Agonist Lixisenatide in Monotherapy: A randomized, double-blind, placebo-controlled trial in patients with type 2 diabetes (GetGoal-Mono) [J]. Diabetes care, 2012, 35(6): 1225-31.
[25] GALLWITZ B, BOHMER M, SEGIET T, et al. Exenatide twice daily versus premixed insulin aspart 70/30 in metformin-treated patients with type 2 diabetes: a randomized 26-week study on glycemic control and hypoglycemia [J]. Diabetes care, 2011, 34(3): 604-6.
[26] GALLWITZ B, GUZMAN J, DOTTA F, et al. Exenatide twice daily versus glimepiride for prevention of glycaemic deterioration in patients with type 2 diabetes with metformin failure (EUREXA): an open-label, randomised controlled trial [J]. The Lancet, 2012, 6736(12): 1-9.
[27] GAO Y, YOON K H, CHUANG L-M, et al. Efficacy and safety of exenatide in patients of Asian descent with type 2 diabetes inadequately controlled with metformin or metformin and a sulphonylurea [J]. Diabetes Res Clin Pract, 2009, 83(1): 69-76.
[28] GARBER A, HENRY R R, RATNER R, et al. Liraglutide, a once-daily human glucagon-like

peptide 1 analogue, provides sustained improvements in glycaemic control and weight for 2 years as monotherapy compared with glimepiride in patients with type 2 diabetes [J]. Diabetes Obes Metab, 2011, 13(4): 348-56.
[29] HEINE R J, VAN GAAL L F, JOHNS D, et al. Exenatide versus insulin glargine in patients with suboptimally controlled type 2 diabetes: a randomized trial [J]. Ann Intern Med, 2005, 143(8): 559-69.
[30] HENRY R R, ROSENSTOCK J, LOGAN D K, et al. Randomized Trial of Continuous Subcutaneous Delivery of Exenatide by ITCA 650 Versus Twice-Daily Exenatide Injections in Metformin-Treated Type 2 Diabetes [J]. Diabetes care, 2013, 36(9): 2559-65.
[31] HOLLANDER P, LASKO B, BARNETT A H, et al. Effects of taspoglutide on glycemic control and body weight in obese patients with type 2 diabetes (T-emerge 7 study) [J]. Obesity (Silver Spring, Md), 2013, 21(2): 238-47.
[32] INAGAKI N, ATSUMI Y, OURA T, et al. Efficacy and safety profile of exenatide once weekly compared with insulin once daily in Japanese patients with type 2 diabetes treated with oral antidiabetes drug(s): results from a 26-week, randomized, open-label, parallel-group, multicenter, noninferiority study [J]. Clin Ther, 2012, 34(9): 1892-908 e1.
[33] IWAMOTO K, NASU R, YAMAMURA A, et al. Safety, tolerability, pharmacokinetics, and pharmacodynamics of exenatide once weekly in Japanese patients with type 2 diabetes [J]. Endocrine Journal, 2009, 56(8): 951-62.
[34] JI L, ONISHI Y, AHN C W, et al. Efficacy and safety of exenatide once-weekly vs exenatide twice-daily in Asian patients with type 2 diabetes mellitus [J]. Journal of Diabetes Investigation, 2013, 4(1): 53-61.
[35] KADOWAKI T, NAMBA M, YAMAMURA A, et al. Exenatide exhibits dose-dependent effects on glycemic control over 12 weeks in Japanese patients with suboptimally controlled type 2 diabetes [J]. Endocrine Journal, 2009, 56(3): 415-24.
[36] KADOWAKI T, NAMBA M, IMAOKA T, et al. Improved glycemic control and reduced bodyweight with exenatide: A double-blind, randomized, phase 3 study in Japanese patients with suboptimally controlled type 2 diabetes over 24 weeks [J]. Journal of Diabetes Investigation, 2011, 2(3): 210-7.
[37] KENDALL D M, RIDDLE M C, ROSENSTOCK J, et al. Effects of exenatide (exendin-4) on glycemic control over 30 weeks in patients with type 2 diabetes treated with metformin and a sulfonylurea [J]. Diabetes care, 2005, 28(5): 1083-91.
[38] KIM D, MACCONELL L, ZHUANG D, et al. Effects of once-weekly dosing of a long-acting release formulation of exenatide on glucose control and body weight in subjects with type 2 diabetes [J]. Diabetes care, 2007, 30(6): 1487-93.
[39] LI C J, LI J, ZHANG Q M, et al. Efficacy and safety comparison between liraglutide as add-on therapy to insulin and insulin dose-increase in Chinese subjects with poorly controlled type 2 diabetes and abdominal obesity [J]. Cardiovascular Diabetology, 2012, 11(
[40] LIUTKUS J, ROSAS GUZMAN J, NORWOOD P, et al. A placebo-controlled trial of exenatide twice-daily added to thiazolidinediones alone or in combination with metformin [J]. Diabetes, Obesity and Metabolism, 2010, 12(12): 1058-65.
[41] MARRE M, SHAW J, BR, et al. Liraglutide, a once-daily human GLP-1 analogue, added to a sulphonylurea over 26 weeks produces greater improvements in glycaemic and weight control compared with adding rosiglitazone or placebo in subjects with Type 2 diabetes (LEAD-1 SU) [J]. Diabetic Medicine, 2009, 26(3): 268-78.

[42] MATHIEU C, RODBARD H W, CARIOU B, et al. A comparison of adding liraglutide versus a single daily dose of insulin aspart to insulin degludec in subjects with type 2 diabetes (BEGIN: VICTOZA ADD-ON) [J]. Diabetes Obes Metab, 2014,
[43] MORETTO T J, MILTON D, X00E, et al. Efficacy and tolerability of exenatide monotherapy over 24 weeks in antidiabetic drug-naive patients with type 2 diabetes: a randomized, double-blind, placebo-controlled, parallel-group study [J]. Clinical Therapeutics, 2008, 30(8): 1448-60.
[44] NAUCK M, WEINSTOCK R S, UMPIERREZ G E, et al. Efficacy and Safety of Dulaglutide Versus Sitagliptin After 52 Weeks in Type 2 Diabetes in a Randomized Controlled Trial (AWARD-5) [J]. Diabetes care, 2014,
[45] NAUCK M A, DURAN S, KIM D, et al. A comparison of twice-daily exenatide and biphasic insulin aspart in patients with type 2 diabetes who were suboptimally controlled with sulfonylurea and metformin: a non-inferiority study [J]. Diabetologia, 2007, 50(2): 259-67.
[46] NAUCK M A, RATNER R E, KAPITZA C, et al. Treatment with the human once-weekly glucagon-like peptide-1 analog taspoglutide in combination with metformin improves glycemic control and lowers body weight in patients with type 2 diabetes inadequately controlled with metformin alone: A double-blind placebo-controlled study [J]. Diabetes care, 2009, 32(7): 1237-43.
[47] NAUCK M, FRID A, HERMANSEN K, et al. Efficacy and safety comparison of liraglutide, glimepiride, and placebo, all in combination with metformin, in type 2 diabetes [J]. Diabetes care, 2009, 32(1): 84-90.
[48] NAUCK M, HORTON E, ANDJELKOVIC M, et al. Taspoglutide, a once-weekly glucagon-like peptide 1 analogue, vs. insulin glargine titrated to target in patients with Type 2 diabetes: an open-label randomized trial [J]. Diabetic medicine : a journal of the British Diabetic Association, 2013, 30(1): 109-13.
[49] The Effect of Liraglutide on Endothelial Function in Subjects With Type 2 Diabetes Mellitus. 2011. http://clinicaltrials.gov/ct2/show/NCT00620282?term=NCT00620282&rank=1 [J].
[50] How Glargine Insulin, Oral Diabetes Medications and Exenatide May Improve Blood Sugar Control and Weight Gain in Type 2 Diabetics. 2013. http://ClinicalTrials.gov/show/NCT00667732 [J].
[51] Effect of Exenatide on Abdominal Fat Distribution in Patients With Type 2 Diabetes Pretreated With Metformin. 2013. [J]. http://ClinicalTrialsgov/show/NCT00701935,
[52] PINGET M, GOLDENBERG R, NIEMOELLER E, et al. Efficacy And Safety Of Lixisenatide Once Daily Versus Placebo In Type 2 Diabetes Insufficiently Controlled On Pioglitazone (Getgoal-P) [J]. Diabetes Obes Metab, 2013, 15(11): 1000-7.
[53] PRATLEY R, NAUCK M, BAILEY T, et al. One year of liraglutide treatment offers sustained and more effective glycaemic control and weight reduction compared with sitagliptin, both in combination with metformin, in patients with type 2 diabetes: A randomised, parallel-group, open-label trial [J]. International Journal of Clinical Practice, 2011, 65(4): 397-407.
[54] PRATLEY R E, UROSEVIC D, BOLDRIN M, et al. Efficacy and tolerability of taspoglutide versus pioglitazone in subjects with type 2 diabetes uncontrolled with sulphonylurea or sulphonylurea-metformin therapy: A randomized, double-blind study (T-emerge 6) [J]. Diabetes, Obesity and Metabolism, 2013, 15(3): 234-40.
[55] PRATLEY R E, NAUCK M A, BARNETT A H, et al. Once-weekly albiglutide versus once-daily liraglutide in patients with type 2 diabetes inadequately controlled on oral drugs (HARMONY 7): a randomised, open-label, multicentre, non-inferiority phase 3 study [J]. The Lancet Diabetes & Endocrinology, 2014, 2(4): 289-97.

[56] RATNER R, NAUCK M, KAPITZA C, et al. Safety and tolerability of high doses of taspoglutide, a once-weekly human GLP-1 analogue, in diabetic patients treated with metformin: A randomized double-blind placebo-controlled study [J]. Diabetic Medicine, 2010, 27(5): 556-62.
[57] RATNER R E, ROSENSTOCK J, BOKA G. Dose-dependent effects of the once-daily GLP-1 receptor agonist lixisenatide in patients with Type 2 diabetes inadequately controlled with metformin: A randomized, double-blind, placebo-controlled trial [J]. Diabetic Medicine, 2010, 27(9): 1024-32.
[58] RAZ I, FONSECA V, KIPNES M, et al. Efficacy and safety of taspoglutide monotherapy in drug-naive type 2 diabetic patients after 24 weeks of treatment: results of a randomized, double-blind, placebo-controlled phase 3 study (T-emerge 1) [J]. Diabetes care, 2012, 35(3): 485-7.
[59] RIDDLE M C, FORST T, ARONSON R, et al. Adding Once-Daily Lixisenatide for Type 2 Diabetes Inadequately Controlled With Newly Initiated and Continuously Titrated Basal Insulin Glargine: A 24-Week, Randomized, Placebo-Controlled Study (GetGoal-Duo 1) [J]. Diabetes care, 2013, 36(9): 2497-503.
[60] RIDDLE M C, ARONSON R, HOME P, et al. Adding Once-Daily Lixisenatide for Type 2 Diabetes Inadequately Controlled by Established Basal Insulin: A 24-week, randomized, placebo-controlled comparison (GetGoal-L) [J]. Diabetes care, 2013, 36(9): 2489-96.
[61] ROSENSTOCK J, BALAS B, CHARBONNEL B, et al. The Fate of Taspoglutide, a Weekly GLP-1 Receptor Agonist, Versus Twice-Daily Exenatide for Type 2 Diabetes: The T-Emerge 2 Trial [J]. Diabetes care, 2013, 36(3): 498-504.
[62] ROSENSTOCK J, HANEFELD M, SHAMANNA P, et al. Beneficial effects of once-daily lixisenatide on overall and postprandial glycemic levels without significant excess of hypoglycemia in type 2 diabetes inadequately controlled on a sulfonylurea with or without metformin (GetGoal-S) [J]. Journal of diabetes and its complications, 2014, 28(3): 386-92.
[63] ROSENSTOCK J, FONSECA V A, GROSS J L, et al. Advancing Basal Insulin Replacement in Type 2 Diabetes Inadequately Controlled With Insulin Glargine Plus Oral Agents: A Comparison of Adding Albiglutide, a Weekly GLP-1 Receptor Agonist, Versus Thrice-Daily Prandial Insulin Lispro [J]. Diabetes care, 2014,
[64] ROSENSTOCK J, REUSCH J, BUSH M, et al. Potential of albiglutide, a long-acting GLP-1 receptor agonist, in type 2 diabetes: a randomized controlled trial exploring weekly, biweekly, and monthly dosing [J]. Diabetes care, 2009, 32(10): 1880-6.
[65] ROSENSTOCK J, RACCAH D, KORANYI L, et al. Efficacy and Safety of Lixisenatide Once Daily Versus Exenatide Twice Daily in Type 2 Diabetes Inadequately Controlled on Metformin: A 24-Week, Randomized, Open-Label, Active-Controlled Study (GetGoal-X) [J]. Diabetes care, 2013,
[66] RUSSELL-JONES D, VAAG A, SCHMITZ O, et al. Liraglutide vs insulin glargine and placebo in combination with metformin and sulfonylurea therapy in type 2 diabetes mellitus (LEAD-5 met+SU): A randomised controlled trial [J]. Diabetologia, 2009, 52(10): 2046-55.
[67] RUSSELL-JONES D, CUDDIHY R M, HANEFELD M, et al. Efficacy and safety of exenatide once weekly versus metformin, pioglitazone, and sitagliptin used as monotherapy in drug-naive patients with type 2 diabetes (DURATION-4): a 26-week double-blind study [J]. Diabetes care, 2012, 35(2): 252-8.
[68] SEINO Y, RASMUSSEN M F, ZDRAVKOVIC M, et al. Dose-dependent improvement in glycemia with once-daily liraglutide without hypoglycemia or weight gain: A double-blind, randomized, controlled trial in Japanese patients with type 2 diabetes [J]. Diabetes Research and Clinical Practice, 2008, 81(2): 161-8.

[69] SEINO Y, RASMUSSEN M F, NISHIDA T, et al. Efficacy and safety of the once-daily human GLP-1 analogue, liraglutide, vs glibenclamide monotherapy in Japanese patients with type 2 diabetes [J]. Current Medical Research and Opinion, 2010, 26(5): 1013-22.
[70] SEINO Y, MIN K W, NIEMOELLER E, et al. Randomized, double-blind, placebo-controlled trial of the once-daily GLP-1 receptor agonist lixisenatide in Asian patients with type 2 diabetes insufficiently controlled on basal insulin with or without a sulfonylurea (GetGoal-L-Asia) [J]. Diabetes Obes Metab, 2012, 14(10): 910-7.
[71] SEINO Y, INAGAKI N, MIYAHARA H, et al. A randomized dose-finding study demonstrating the efficacy and tolerability of albiglutide in Japanese patients with type 2 diabetes mellitus [J]. Curr Med Res Opin, 2014, 30(6): 1095-106.
[72] UMPIERREZ G, POVEDANO S T, MANGHI F P, et al. Efficacy and Safety of Dulaglutide Monotherapy Versus Metformin in Type 2 Diabetes in a Randomized Controlled Trial (AWARD-3) [J]. Diabetes care, 2014,
[73] UMPIERREZ G E, BLEVINS T, ROSENSTOCK J, et al. The effects of LY2189265, a long-acting glucagon-like peptide-1 analogue, in a randomized, placebo-controlled, double-blind study of overweight/obese patients with type 2 diabetes: the EGO study [J]. Diabetes Obes Metab, 2011, 13(5): 418-25.
[74] WYSHAM C, BLEVINS T, ARAKAKI R, et al. Efficacy and Safety of Dulaglutide Added on to Pioglitazone and Metformin Versus Exenatide in Type 2 Diabetes in a Randomized Controlled Trial (AWARD-1) [J]. Diabetes care, 2014,
[75] YANG W, CHEN L, JI Q, et al. Liraglutide provides similar glycaemic control as glimepiride (both in combination with metformin) and reduces body weight and systolic blood pressure in Asian population with type 2 diabetes from China, South Korea and India: A 16-week, randomized, double-blind, active control trial [J]. Diabetes, Obesity and Metabolism, 2011, 13(1): 81-8.
[76] YUAN G H, SONG W L, HUANG Y Y, et al. Efficacy and tolerability of exenatide monotherapy in obese patients with newly diagnosed type 2 diabetes: A randomized, 26 weeks metformin-controlled, parallel-group study [J]. Chinese Medical Journal, 2012, 125(15): 2677-81.
[77] ZINMAN B, HOOGWERF B J, DURAN GARCIA S, et al. The effect of adding exenatide to a thiazolidinedione in suboptimally controlled type 2 diabetes: a randomized trial [J]. Ann Intern Med, 2007, 146(7): 477-85.
[78] ZINMAN B, GERICH J, BUSE J B, et al. Efficacy and safety of the human glucagon-like peptide-1 analog liraglutide in combination with metformin and thiazolidinedione in patients with type 2 diabetes (LEAD-4 Met+TZD).[Erratum appears in Diabetes Care. 2010 Mar;33(3):692] [J]. Diabetes care, 2009, 32(7): 1224-30.
